# A Framework for Susceptibility Analysis of Brain Tumours Based on Uncertain Analytical Cum Algorithmic Modeling

**DOI:** 10.3390/bioengineering10020147

**Published:** 2023-01-22

**Authors:** Atiqe Ur Rahman, Muhammad Saeed, Muhammad Haris Saeed, Dilovan Asaad Zebari, Marwan Albahar, Karrar Hameed Abdulkareem, Alaa S. Al-Waisy, Mazin Abed Mohammed

**Affiliations:** 1 Department of Mathematics, University of Management and Technology, Lahore 54000, Pakistan; 2 Department of Chemistry, University of Management and Technology, Lahore 54000, Pakistan; 3 Department of Computer Science, College of Science, Nawroz University, Duhok 42001, Iraq; 4 School of Computer Science, Umm Al Qura University, Mecca 24211, Saudi Arabia; 5 College of Agriculture, Al-Muthanna University, Samawah 66001, Iraq; 6 Computer Technologies Engineering Department, Information Technology College, Imam Ja’afar Al-Sadiq University, Baghdad 10001, Iraq; 7 College of Computer Science and Information Technology, University of Anbar, Anbar 31001, Iraq

**Keywords:** brain tumour, complex intuitionistic fuzzy set, core matrix, fuzzy parameterisation, hypersoft set, susceptibility analysis

## Abstract

Susceptibility analysis is an intelligent technique that not only assists decision makers in assessing the suspected severity of any sort of brain tumour in a patient but also helps them diagnose and cure these tumours. This technique has been proven more useful in those developing countries where the available health-based and funding-based resources are limited. By employing set-based operations of an arithmetical model, namely fuzzy parameterised complex intuitionistic fuzzy hypersoft set (FPCIFHSS), this study seeks to develop a robust multi-attribute decision support mechanism for appraising patients’ susceptibility to brain tumours. The FPCIFHSS is regarded as more reliable and generalised for handling information-based uncertainties because its complex components and fuzzy parameterisation are designed to deal with the periodic nature of the data and dubious parameters (sub-parameters), respectively. In the proposed FPCIFHSS-susceptibility model, some suitable types of brain tumours are approximated with respect to the most relevant symptoms (parameters) based on the expert opinions of decision makers in terms of complex intuitionistic fuzzy numbers (CIFNs). After determining the fuzzy parameterised values of multi-argument-based tuples and converting the CIFNs into fuzzy values, the scores for such types of tumours are computed based on a core matrix which relates them with fuzzy parameterised multi-argument-based tuples. The sub-intervals within [0, 1] denote the susceptibility degrees of patients corresponding to these types of brain tumours. The susceptibility of patients is examined by observing the membership of score values in the sub-intervals.

## 1. Introduction

Over the course of time, human intelligence coupled with advancements in biomedical systems have allowed for the development of methodologies and procedures that allow medical professionals to access cancerous masses in a more in-depth manner and more concisely. However, these advancements still fall short when considering curing methods of cancerous tumours [1]. Multiple avenues of research can be generated with a single research question while following different methodologies for solving that problem with unique analytical pipelines. So, when considering the field of functional neuroimaging coupled with the development of diagnostic systems in its fledgling nature, analytical exploration is an inescapable process of the scientific method and can lead to significant discoveries over time.

The brain is the most complex organ in the human body and even a slight abnormality can have catastrophic effects on the whole body. The statistics indicate that about 23,000 people fell prey to cancerous tumours in the brain in the USA alone, indicating it as one of the major emerging ailments [2]. The cancer indicator reports indicate the probability of development of these tumours is the same for adults and children [3]. Another report indicated that a total of 80,000 new cases of brain tumours were reported in 2018, which were divided into four classes based on their location in the brain: meningioma represented 36.3% (29,320), gliomas 26.5% (21,200), pituitary tumours represented nearly 16.2% (13,210) and the rest of the cases belonged to other types of brain tumour such as malignant, medulloblastoma and lymphomas [4]. The majority of the cases of reported meningiomas start as benign lesions from a histological perspective [5]. Based on these figures, the timely diagnosis and effective handling of a tumour are essential for the patient. Advancements in the field of medical and neural imaging had allowed for timely detection of these lesions in the pre-symptomatic stages, unlike a couple of decades ago when these tumours were only detected when they became large and severely symptomatic [6]. When studying these tumours, some of them never became symptomatic while others progressed to cause symptoms, raising the question of which patient to select for treatment and what treatment methods to opt for for optimal results [7,8,9]. In the case of most tumours, surgery is the very first option for tumours with large size, while for those masses that are close to radiosensitive structures like the optic apparatus, SRS is a viable option for the treatment of small meningiomas [10,11,12].

Now, the mode of therapy to choose for effective treatment of the tumour relies on the pathological nature of the tumour, the stage at which it is diagnosed and the tumour category. When diagnosing a patient with a particular disease, medical imaging techniques and the intuition of medical professionals go hand in hand. The diagnostic process is highly reliant on how the medical images are perceived by the medical professionals while considering other symptomatic conditions simultaneously. Medical professionals use computer-aided diagnosis (CAD) to efficiently analyse and classify pathological and imaging data obtained for diagnosing brain tumours [13]. With recent advances in machine learning, data mining and artificial intelligence, these CAD models have come a long way. In the case of brain tumour diagnosis, the accuracy of these models is still sub-par for regular medical use. Researchers are trying to improve the accuracy of these models by processing huge data sets of images and diagnostic test data of brain tumours using deep learning models for improvement in diagnostic accuracy [14,15]. Another approach that is on par with these models is applying fuzzy set theory concepts to design diagnostic support systems. When diagnosing a patient with a particular disease, medical imaging techniques and the intuition of medical professionals go hand in hand. The diagnostic process is highly reliant on how the medical images are perceived by the medical professionals while considering other symptomatic conditions at the same time. So, this diagnostic process presents itself as an MADM (a particular type of MCDM) problem where the medical professional decides, based on the numerous factors of different natures, to come to a tentative decision in the form of a diagnosis. Fuzzy set theory has been extensively used in designing decision support systems as it is considered to handle human intuition using mathematical syntax. The idea was introduced by Zadeh [16]. This theory became the basis of numerous other decision-making studies and led to the development of complex hybrid mathematical structures such as the concept of rough set and period mathematics.

As an extension of existing structures [17,18,19,20], Smarandache put forward the concept of hypersoft set (HSS) [21] which is an emerging field of research to tackle data-based vagueness and uncertainties. Recently, Saeed et al. [22] presented various set-based operations of HSS. The HSS has been a subject of great interest for many researchers since its introduction in 2018. Its hybridised models have been applied in many multidisciplinary areas while dealing with MADM problems. Saeed et al. [23,24,25,26] studied the diagnoses of diseases such as COVID-19, tuberculosis, allergy-based diseases, and hepatitis, respectively, by using the hybrids of HSS. Similarly, Rahman et al. [27,28] studied the diagnosis of heart disease by proposing robust algorithmic MADM techniques based on hybrids of HSS.

Analysis of susceptibility to brain tumours has attracted many researchers but the most significant contributions are the following: Romano et al. [29] discussed clinical applications of dynamic susceptibility contrast perfusion-weighted MR imaging in brain tumours. Järnum et al. [30] investigated the perfusion MRI of brain tumours by providing a comparative study of pseudo-continuous arterial spin labeling and dynamic susceptibility contrast imaging. Fayed et al. [31] proposed a robust technique for malignancy assessment of brain tumours with magnetic resonance spectroscopy and dynamic susceptibility contrast MRI. Lehmann et al. [32] presented the comparative study of perfusion measurement in brain tumours at 3 Tesla MR on the basis of arterial spin labeling versus dynamic susceptibility contrast-enhanced MRI. Park et al. [33] combined the high-resolution susceptibility-weighted imaging and the apparent diffusion coefficient by assigning value to brain tumour imaging and clinical feasibility of non-contrast MRI at 3T. The majority of the studies involve the application of fuzzy hybrid structures with reference to brain tumours that employ fuzzy logic to classify brain tumours based on data obtained from neuroimaging techniques. Some examples include the use of a picture fuzzy clustering method for segmentations of brain MRI images [34], tumour symmetry analysis using spatially constrained deformable models based on fuzzy classification of 3D MRI images [35], classification of brain tumour type using fuzzy cognitive maps [36] and studies involving the concept of hypersoft mapping for the suggestion of an appropriate treatment method based on the type of tumour [37].

Although the research work conducted by Saeed et al. [37] regarding the diagnosis of brain tumours is convincing, it is not sufficient for real-world MADM situations such as a medical diagnosis in which the regular data-based periodicity is observed. Similarly, they employed the concept hypersoft mapping with an indeterminate setting with many computation-based complexities. As the work of Saeed et al. is the research item most relevant to this study, the scarcity of such research motivates the authors to initiate this paper. The proposed structure FPCIFHSS is more flexible and easily understandable as compared to the mentioned studies. It has a lesser degree of computational complexities, which enables readers from multidisciplinary fields of study to understand its findings and computations with great ease.

The prominent contributions of the paper can be outlined as follows:

1. The ambiguous nature of parameters (symptoms) and their related sub-parameters are managed by applying the concept of fuzzy parameterisation, which assigns them a fuzzy membership grade. An innovative algebraic method, namely the FPCIFHSS-Ranking method, is used to find out the fuzzy parameterised values of parametric valued tuples based on their complex intuitionistic fuzzy numbers-based approximation. The data-based periodicity is treated with the use of amplitude and phase values (complex plane setting). The amplitude value is meant for the membership magnitude and the other is for its periodic value.
2. A novel robust MADM-based algorithm i.e., Pythagorean Means-based Scoring Algorithm (PMBSA) is proposed by using set-based operations of FPCIFHSS to assess analyses of the susceptibility of patients to brain tumours. These steps of the proposed algorithms are easily understandable and free of computational complexities.
3. The expert opinions of decision makers (medical experts with expertise in dealing with brain tumours such as neurologists, neuropathologists, internists, oncologists, radiation oncologists, neuro-oncologists and neurosurgeons) are gathered in terms of CIFNs, which are not only easily computable but also easily transformable to fuzzy values. The patients are first assigned a susceptibility degree in subintervals within [0, 1] then scores of brain tumours, computed from the proposed algorithm, are matched on their respective

As far as the organisation of the remainder of this paper is concerned, the next section (Section 2) consists of some preliminary knowledge which is provided to understand the main methodology and the presented mathematical structure with great ease. Section 3 describes the various stages of the proposed methodology, including the characterisation of the basic notions of FPCIFHSS, the role of fuzzy parameterisation for the symptoms and their related subcategories, arithmetic criteria for the transformation of CIFNs to CFNs and then CFNs to fuzzy values, the process for the selection of parameters and sub-parameters and the profiles of decision makers for this evaluation. It also includes an MADM system based on the proposal of a robust algorithm for assessing the susceptibility of patients to brain tumours using aggregation-based operations of FPCIFHSS. Section 4 presents the discussion, sensitivity analysis and comparison of the proposed study. Finally, Section 5 concludes the paper with limitations and future scope.

### Abbreviations and Acronyms

All the abbreviations and acronyms (that are used in the paper) along with their full names are presented in Table 1.

## 2. Preliminary Knowledge

This section reviews some preliminary definitions to assist the main results. The following applies throughout the remainder of the paper.

**Definition 1**  (Atanassov, [17]). *An intuitionistic fuzzy set $\Psi^{\hat{\Lambda }}$ over $\hat{\Lambda }$ is stated as*

(1) $$\Psi^{\hat{\Lambda }}=\left\{\left(\hat{a},\langle {\hat{T}}_{\Psi }\left(\hat{a}\right),{\hat{F}}_{\Psi }\left(\hat{a}\right)\rangle \right)|\;\hat{a}\in \hat{\Lambda }\right\}w$$

*here ${\hat{T}}_{\Psi },{\hat{F}}_{\Psi }:\hat{\Lambda }\to \left[0,1\right]$ such that $\left({\hat{T}}_{\Psi }\left(\hat{a}\right)+{\hat{F}}_{\Psi }\left(\hat{a}\right)\right)\in \left[0,1\right]$ and the values ${\hat{T}}_{\Psi }\left(\hat{a}\right)$ and ${\hat{F}}_{\Psi }\left(\hat{a}\right)$ are true and false belonging grades of $\hat{a}$, respectively. The grade of hesitancy is ${\hat{H}}_{\Psi }\left(\hat{a}\right)=1-{\hat{T}}_{\Psi }\left(\hat{a}\right)-{\hat{F}}_{\Psi }\left(\hat{a}\right)$.*

**Example 1.**  *Let $\hat{\Lambda }=\left\{{\hat{a}}_{1},{\hat{a}}_{2},{\hat{a}}_{3},{\hat{a}}_{4}\right\}$ be a collection of objects with ${\hat{T}}_{\Psi }\left(\hat{x_{1}}\right)=0.4$, ${\hat{T}}_{\Psi }\left(\hat{x_{2}}\right)=0.3$, ${\hat{T}}_{\Psi }\left(\hat{x_{3}}\right)=0.2$, ${\hat{T}}_{\Psi }\left(\hat{x_{4}}\right)=0.6$, ${\hat{F}}_{\Psi }\left(\hat{x_{1}}\right)=0.3$, ${\hat{F}}_{\Psi }\left(\hat{x_{2}}\right)=0.5$, ${\hat{F}}_{\Psi }\left(\hat{x_{3}}\right)=0.7$ and ${\hat{F}}_{\Psi }\left(\hat{x_{4}}\right)=0.2$; then an IFS $\Psi^{\hat{\Lambda }}$ is constructed as*

$$\Psi^{\hat{\Lambda }}=\left\{\begin{array}{c} \left({\hat{a}}_{1},\langle 0.4,0.3\rangle \right),\left({\hat{a}}_{2},\langle 0.3,0.5\rangle \right),\left({\hat{a}}_{3},\langle 0.2,0.7\rangle \right),\left({\hat{a}}_{4},\langle 0.6,0.2\rangle \right) \end{array}\right\}$$

*with hesitancy grades ${\hat{H}}_{\Psi }\left({\hat{a}}_{1}\right)=0.3$, ${\hat{H}}_{\Psi }\left({\hat{a}}_{2}\right)=0.2$, ${\hat{H}}_{\Psi }\left({\hat{a}}_{3}\right)=0.1$ and ${\hat{H}}_{\Psi }\left({\hat{a}}_{4}\right)=0.2$.*

**Definition 2**  (Alkouri, [19]). *A complex intuitionistic fuzzy set $\zeta^{\hat{\Lambda }}$ over $\hat{\Lambda }$ is stated as*

(2) $$\zeta^{\hat{\Lambda }}=\left\{\left(\hat{a},\langle {\hat{T}}_{\zeta }\left(\hat{a}\right),{\hat{F}}_{\zeta }\left(\hat{a}\right)\rangle \right)|\;\hat{a}\in \hat{\Lambda }\right\}w$$

*where ${\hat{T}}_{\zeta }={\hat{A}}_{\hat{T}}\;e^{j{\hat{B}}_{\hat{T}}}$, ${\hat{F}}_{\zeta }={\hat{A}}_{\hat{F}}\;e^{j{\hat{B}}_{\hat{F}}}$ are complex valued true and false belonging mappings such that $\left({\hat{A}}_{\hat{T}}\left(\hat{a}\right)+{\hat{A}}_{\hat{F}}\left(\hat{a}\right)\right)\in \left[0,1\right]$ and $\left({\hat{B}}_{\hat{T}}\left(\hat{a}\right)+{\hat{B}}_{\hat{F}}\left(\hat{a}\right)\right)\in \left[0,2\pi \right]$. The values ${\hat{A}}_{\hat{T}}\left(\hat{a}\right)$ and ${\hat{A}}_{\hat{F}}\left(\hat{a}\right)$ are the amplitude terms for true and false belonging grades of $\hat{a}$, respectively. Similarly, the values ${\hat{B}}_{\hat{T}}\left(\hat{a}\right)$ and ${\hat{B}}_{\hat{F}}\left(\hat{a}\right)$ are the phase terms for true and false belonging grades of $\hat{a}$, respectively. The grades of hesitancy for amplitude and phase terms are ${\hat{H}}_{amp}\left(\hat{a}\right)=1-{\hat{A}}_{\hat{T}}\left(\hat{a}\right)-{\hat{A}}_{\hat{F}}\left(\hat{a}\right)$ and ${\hat{H}}_{pha}\left(\hat{a}\right)=2\pi -{\hat{B}}_{\hat{T}}\left(\hat{a}\right)-{\hat{B}}_{\hat{F}}\left(\hat{a}\right)$.*

**Example 2.**  *Let $\hat{\Lambda }=\left\{{\hat{a}}_{1},{\hat{a}}_{2},{\hat{a}}_{3},{\hat{a}}_{4}\right\}$ be a collection of objects with ${\hat{T}}_{\zeta }\left(\hat{x_{1}}\right)=0.4\;e^{j2\pi (0.2)}$, ${\hat{T}}_{\zeta }\left(\hat{x_{2}}\right)=0.3\;e^{j2\pi (0.1)}$, ${\hat{T}}_{\zeta }\left(\hat{x_{3}}\right)=0.2\;e^{j2\pi (0.4)}$, ${\hat{T}}_{\zeta }\left(\hat{x_{4}}\right)=0.6\;e^{j2\pi (0.3)}$, ${\hat{F}}_{\zeta }\left(\hat{x_{1}}\right)=0.3\;e^{j2\pi (0.6)}$, ${\hat{F}}_{\zeta }\left(\hat{x_{2}}\right)=0.5\;e^{j2\pi (0.5)}$, ${\hat{F}}_{\zeta }\left(\hat{x_{3}}\right)=0.7\;e^{j2\pi (0.4)}$ and ${\hat{F}}_{\zeta }\left(\hat{x_{4}}\right)=0.2\;e^{j2\pi (0.3)}$; then a CIFS $\zeta^{\hat{\Lambda }}$ is constructed as*

$$\zeta^{\hat{\Lambda }}=\left\{\begin{array}{c} \left({\hat{a}}_{1},\langle 0.4\;e^{j2\pi (0.2)},0.3\;e^{j2\pi (0.6)}\rangle \right),\left({\hat{a}}_{2},\langle 0.3\;e^{j2\pi (0.1)},0.5\;e^{j2\pi (0.5)}\rangle \right),\\ \left({\hat{a}}_{3},\langle 0.2\;e^{j2\pi (0.4)},0.7\;e^{j2\pi (0.4)}\rangle \right),\left({\hat{a}}_{4},\langle 0.6\;e^{j2\pi (0.3)},0.2\;e^{j2\pi (0.3)}\rangle \right) \end{array}\right\}$$

*with hesitancy grades ${\hat{H}}_{amp}\left({\hat{a}}_{1}\right)=0.3$, ${\hat{H}}_{amp}\left({\hat{a}}_{2}\right)=0.2$, ${\hat{H}}_{amp}\left({\hat{a}}_{3}\right)=0.1$, ${\hat{H}}_{amp}\left({\hat{a}}_{4}\right)=0.2$, ${\hat{H}}_{pha}\left({\hat{a}}_{1}\right)=2\pi \left(0.2\right)$, ${\hat{H}}_{pha}\left({\hat{a}}_{2}\right)=2\pi \left(0.4\right)$, ${\hat{H}}_{pha}\left({\hat{a}}_{3}\right)=2\pi \left(0.2\right)$ and ${\hat{H}}_{pha}\left({\hat{a}}_{4}\right)=2\pi \left(0.4\right)$.*

**Definition 3**  (Molodtsov, [20]). *Let $\hat{\Xi }$ and $\hat{\Lambda }$ be the sets consisting of evaluating features (attributes) and alternatives, respectively. An SS $\Gamma^{\hat{\Lambda }}$ over $\hat{\Lambda }$ is stated as*

(3) $$\Gamma^{\hat{\Lambda }}=\left\{\left(\hat{e},\xi_{\Gamma }\left(\hat{e}\right)\right)|\;\xi_{\Gamma }\left(\hat{e}\right)\subseteq \hat{\Lambda }\;\&\;\hat{e}\in \hat{\Xi }\right\}$$

*in which $\xi_{\Gamma }:\hat{\Xi }\to 2^{\hat{\Lambda }}$ is an approximate mapping and the value $\xi_{\Gamma }\left(\hat{e}\right)$ is an $\hat{e}$-approximate entity of $\Gamma^{\hat{\Lambda }}$.*

**Definition 4**  (Smarandache, [21], Rahman et al. [27]). *Let ${\hat{\Xi }}_{i}$ be the sets containing the sub-parametric values of the parameters ${\hat{e}}_{i}$ belonging to $\hat{\Xi }$ such that for any two ${\hat{e}}_{i}\neq {\hat{e}}_{j}$, the corresponding sets ${\hat{\Xi }}_{i}$ and ${\hat{\Xi }}_{j}$ are non-overlapping. The HSS ${\hat{\hbar }}^{\hat{\Lambda }}$ over $\hat{\Lambda }$ is stated as*

(4) $${\hat{\hbar }}^{\hat{\Lambda }}=\left\{\left(\hat{℘},\xi_{\hat{\hbar }}\left(\hat{℘}\right)\right)|\;\xi_{\hat{\hbar }}\left(\hat{℘}\right)\subseteq \hat{\Lambda }\;\&\;\hat{℘}\in \hat{ℑ}\right\}i$$

*n which $\xi_{\hat{\hbar }}:\hat{ℑ}\to 2^{\hat{\Lambda }}$ is a multi argument approximate mapping, $\hat{ℑ}=\prod_{i}{\hat{\Xi }}_{i}$ and the value $\xi_{\hat{\hbar }}\left(\hat{℘}\right)$ is a $\hat{℘}$-multi-approximate entity of ${\hat{\hbar }}^{\hat{\Lambda }}$.*

## 3. Salient Features of Proposed Methodology

**Figure 1:**
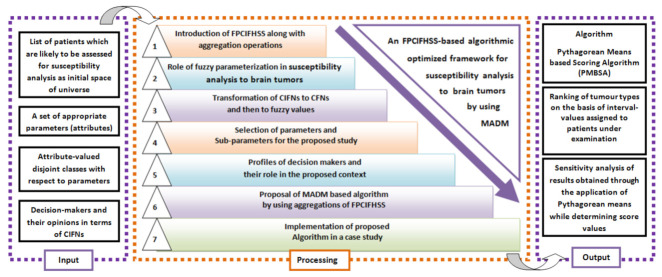
The brief pictorial description of proposed decision support framework.

This section discusses the various aspects of stages involved in the proposed methodology. In the first stage, a novel algebraic model, i.e., fuzzy parameterised complex intuitionistic fuzzy hypersoft set (FPCIFHSS), is introduced, which best suits the analysis of susceptibility to brain tumours by considering its expected uncertainties. The stages of the adopted methodology are presented briefly in Figure 1.

### 3.1. The FPCIFHSS and Its Set-Theoretic Operations

In this part, the definition of FPCIFHSS and its aggregation operations are presented with examples.

**Definition 5.**  *Let ${\hat{\Xi }}_{i}$ be the sets containing the sub-parametric values of the parameters ${\hat{e}}_{i}$ belonging to $\hat{\Xi }$ such that for any two ${\hat{e}}_{i}\neq {\hat{e}}_{j}$, the corresponding sets ${\hat{\Xi }}_{i}$ and ${\hat{\Xi }}_{j}$ are non-overlapping and for ${\hat{℘}}_{k}\in \hat{ℑ}=\prod_{i}{\hat{\Xi }}_{i}$ where k is the total number of elements in $\hat{ℑ}$, $\hat{F}=\left\{\frac{{\hat{℘}}_{k}}{\hat{\psi }\left({\hat{℘}}_{k}\right)}:\hat{\psi }\left({\hat{℘}}_{k}\right)\in \left[0,1\right]\right\}$ is a fuzzy set over $\hat{ℑ}$, then an FPCIFHSS ${\hat{\Theta }}^{\hat{\Lambda }}$ over $\hat{\Lambda }$ can be stated as*

(5) $${\hat{\Theta }}^{\hat{\Lambda }}=\left\{\left(\frac{{\hat{℘}}_{k}}{\hat{\psi }\left({\hat{℘}}_{k}\right)},\Psi \left(\frac{{\hat{℘}}_{k}}{\hat{\psi }\left({\hat{℘}}_{k}\right)}\right)\right)|\;{\hat{a}}_{i}\in \hat{\Lambda },\frac{{\hat{℘}}_{k}}{\hat{\psi }\left({\hat{℘}}_{k}\right)}\in \hat{F}\right\}$$

*where $\Psi \left(\frac{{\hat{℘}}_{k}}{\hat{\psi }\left({\hat{℘}}_{k}\right)}\right)=\langle {\hat{T}}_{\hat{\Theta }}\left(\frac{{\hat{℘}}_{k}}{\hat{\psi }\left({\hat{℘}}_{k}\right)}\right)\left({\hat{a}}_{i}\right),{\hat{F}}_{\hat{\Theta }}\left(\frac{{\hat{℘}}_{k}}{\hat{\psi }\left({\hat{℘}}_{k}\right)}\right)\left({\hat{a}}_{i}\right)\rangle$ with*

(6) $${\hat{T}}_{\hat{\Theta }}\left(\frac{{\hat{℘}}_{k}}{\hat{\psi }\left({\hat{℘}}_{k}\right)}\right)\left({\hat{a}}_{i}\right)={\hat{A}}_{\hat{T}}\left(\frac{{\hat{℘}}_{k}}{\hat{\psi }\left({\hat{℘}}_{k}\right)}\right)\left({\hat{a}}_{i}\right)\;e^{j{\hat{B}}_{\hat{T}}\left(\frac{{\hat{℘}}_{k}}{\hat{\psi }\left({\hat{℘}}_{k}\right)}\right)\left({\hat{a}}_{i}\right)}$$

(7) $${\hat{F}}_{\hat{\Theta }}\left(\frac{{\hat{℘}}_{k}}{\hat{\psi }\left({\hat{℘}}_{k}\right)}\right)\left({\hat{a}}_{i}\right)={\hat{A}}_{\hat{F}}\left(\frac{{\hat{℘}}_{k}}{\hat{\psi }\left({\hat{℘}}_{k}\right)}\right)\left({\hat{a}}_{i}\right)\;e^{j{\hat{B}}_{\hat{F}}\left(\frac{{\hat{℘}}_{k}}{\hat{\psi }\left({\hat{℘}}_{k}\right)}\right)\left({\hat{a}}_{i}\right)}$$

*are complex valued true and false belonging mappings such that $\left({\hat{A}}_{\hat{T}}\left(\frac{{\hat{℘}}_{k}}{\hat{\psi }\left({\hat{℘}}_{k}\right)}\right)\left({\hat{a}}_{i}\right)+{\hat{A}}_{\hat{F}}\left(\frac{{\hat{℘}}_{k}}{\hat{\psi }\left({\hat{℘}}_{k}\right)}\right)\left({\hat{a}}_{i}\right)\right)\in \left[0,1\right]$ and $\left({\hat{B}}_{\hat{T}}\left(\frac{{\hat{℘}}_{k}}{\hat{\psi }\left({\hat{℘}}_{k}\right)}\right)\left({\hat{a}}_{i}\right)+{\hat{B}}_{\hat{F}}\left(\frac{{\hat{℘}}_{k}}{\hat{\psi }\left({\hat{℘}}_{k}\right)}\right)\left({\hat{a}}_{i}\right)\right)\in \left[0,2\pi \right]$. The values ${\hat{A}}_{\hat{T}}\left(\frac{{\hat{℘}}_{k}}{\hat{\psi }\left({\hat{℘}}_{k}\right)}\right)\left({\hat{a}}_{i}\right)$ and ${\hat{A}}_{\hat{F}}\left(\frac{{\hat{℘}}_{k}}{\hat{\psi }\left({\hat{℘}}_{k}\right)}\right)$$\left({\hat{a}}_{i}\right)$ are the amplitude terms for true and false belonging grades of ${\hat{a}}_{i}$, respectively. Similarly, the values ${\hat{B}}_{\hat{T}}\left({\hat{a}}_{i}\right)$ and ${\hat{B}}_{\hat{F}}\left(\frac{{\hat{℘}}_{k}}{\hat{\psi }\left({\hat{℘}}_{k}\right)}\right)\left({\hat{a}}_{i}\right)$ are the phase terms for true and false belonging grades of ${\hat{a}}_{i}$, respectively. The grades of hesitancy for amplitude and phase terms are ${\hat{H}}_{amp}\left(\frac{{\hat{℘}}_{k}}{\hat{\psi }\left({\hat{℘}}_{k}\right)}\right)\left({\hat{a}}_{i}\right)=1-{\hat{A}}_{\hat{T}}\left(\frac{{\hat{℘}}_{k}}{\hat{\psi }\left({\hat{℘}}_{k}\right)}\right)\left({\hat{a}}_{i}\right)-{\hat{A}}_{\hat{F}}\left(\frac{{\hat{℘}}_{k}}{\hat{\psi }\left({\hat{℘}}_{k}\right)}\right)\left({\hat{a}}_{i}\right)$ and ${\hat{H}}_{pha}\left(\frac{{\hat{℘}}_{k}}{\hat{\psi }\left({\hat{℘}}_{k}\right)}\right)\left({\hat{a}}_{i}\right)=2\pi -{\hat{B}}_{\hat{T}}\left(\frac{{\hat{℘}}_{k}}{\hat{\psi }\left({\hat{℘}}_{k}\right)}\right)\left({\hat{a}}_{i}\right)-{\hat{B}}_{\hat{F}}\left(\frac{{\hat{℘}}_{k}}{\hat{\psi }\left({\hat{℘}}_{k}\right)}\right)\left({\hat{a}}_{i}\right)$.*

**Example 3.**  *Let $\hat{\Lambda }=\left\{{\hat{a}}_{1},{\hat{a}}_{2},{\hat{a}}_{3},{\hat{a}}_{4}\right\}$ be a collection of objects, $\hat{\Xi }=\left\{{\hat{e}}_{1},{\hat{e}}_{2},{\hat{e}}_{3}\right\}$ be the set of parameters and the respective parametric valued non-overlapping sets be ${\hat{\Xi }}_{1}=\left\{{\hat{e}}_{11},{\hat{e}}_{12}\right\}$, ${\hat{\Xi }}_{2}=\left\{{\hat{e}}_{21},{\hat{e}}_{22}\right\}$ and ${\hat{\Xi }}_{3}=\left\{{\hat{e}}_{31}\right\}$, respectively, such that $\hat{ℑ}=\prod_{i=1}^{3}{\hat{\Xi }}_{i}=\left\{{\hat{℘}}_{1}=\left({\hat{e}}_{11},{\hat{e}}_{21},{\hat{e}}_{31}\right),{\hat{℘}}_{2}=\left({\hat{e}}_{11},{\hat{e}}_{22},{\hat{e}}_{31}\right),{\hat{℘}}_{3}=\left({\hat{e}}_{12},{\hat{e}}_{21},{\hat{e}}_{31}\right),{\hat{℘}}_{4}=\left({\hat{e}}_{21},{\hat{e}}_{22},{\hat{e}}_{31}\right)\right\}$, then the fuzzy set $\hat{F}$ over $\hat{ℑ}$ is $\{\frac{{\hat{℘}}_{1}}{0.1},\frac{{\hat{℘}}_{2}}{0.3},\frac{{\hat{℘}}_{3}}{0.5},$$\frac{{\hat{℘}}_{4}}{0.6}\}$, then FPCIFHSS ${\hat{\Theta }}^{\hat{\Lambda }}$ over $\hat{\Lambda }$ can be constructed as*

$${\hat{\Theta }}^{\hat{\Lambda }}=\left\{\begin{array}{c} \left(\frac{{\hat{℘}}_{1}}{0.1},\left\{\begin{array}{c} \left({\hat{a}}_{1},\langle 0.2\;e^{j2\pi (0.3)},0.3\;e^{j2\pi (0.4)}\rangle \right),\left({\hat{a}}_{2},\langle 0.4\;e^{j2\pi (0.5)},0.5\;e^{j2\pi (0.6)}\rangle \right),\\ \left({\hat{a}}_{3},\langle 0.6\;e^{j2\pi (0.7)},0.3\;e^{j2\pi (0.8)}\rangle \right),\left({\hat{a}}_{4},\langle 0.8\;e^{j2\pi (0.4)},0.1\;e^{j2\pi (0.6)}\rangle \right) \end{array}\right\}\right),\\ \left(\frac{{\hat{℘}}_{2}}{0.3},\left\{\begin{array}{c} \left({\hat{a}}_{1},\langle 0.7\;e^{j2\pi (0.4)},0.1\;e^{j2\pi (0.3)}\rangle \right),\left({\hat{a}}_{2},\langle 0.6\;e^{j2\pi (0.5)},0.2\;e^{j2\pi (0.3)}\rangle \right),\\ \left({\hat{a}}_{3},\langle 0.5\;e^{j2\pi (0.6)},0.2\;e^{j2\pi (0.5)}\rangle \right),\left({\hat{a}}_{4},\langle 0.4\;e^{j2\pi (0.4)},0.3\;e^{j2\pi (0.5)}\rangle \right) \end{array}\right\}\right),\\ \left(\frac{{\hat{℘}}_{3}}{0.5},\left\{\begin{array}{c} \left({\hat{a}}_{1},\langle 0.8\;e^{j2\pi (0.6)},0.1\;e^{j2\pi (0.5)}\rangle \right),\left({\hat{a}}_{2},\langle 0.7\;e^{j2\pi (0.5)},0.2\;e^{j2\pi (0.4)}\rangle \right),\\ \left({\hat{a}}_{3},\langle 0.6\;e^{j2\pi (0.6)},0.1\;e^{j2\pi (0.1)}\rangle \right),\left({\hat{a}}_{4},\langle 0.5\;e^{j2\pi (0.3)},0.3\;e^{j2\pi (0.6)}\rangle \right) \end{array}\right\}\right),\\ \left(\frac{{\hat{℘}}_{4}}{0.6},\left\{\begin{array}{c} \left({\hat{a}}_{1},\langle 0.5\;e^{j2\pi (0.1)},0.1\;e^{j2\pi (0.1)}\rangle \right),\left({\hat{a}}_{2},\langle 0.4\;e^{j2\pi (0.2)},0.3\;e^{j2\pi (0.2)}\rangle \right),\\ \left({\hat{a}}_{3},\langle 0.3\;e^{j2\pi (0.1)},0.1\;e^{j2\pi (0.5)}\rangle \right),\left({\hat{a}}_{4},\langle 0.2\;e^{j2\pi (0.6)},0.4\;e^{j2\pi (0.6)}\rangle \right) \end{array}\right\}\right) \end{array}\right\}$$

*with hesitancy grades ${\hat{H}}_{amp}\left({\hat{a}}_{1}\right)=0.3$, ${\hat{H}}_{amp}\left({\hat{a}}_{2}\right)=0.2$, ${\hat{H}}_{amp}\left({\hat{a}}_{3}\right)=0.1$, ${\hat{H}}_{amp}\left({\hat{a}}_{4}\right)=0.2$, ${\hat{H}}_{pha}\left({\hat{a}}_{1}\right)=2\pi \left(0.2\right)$, ${\hat{H}}_{pha}\left({\hat{a}}_{2}\right)=2\pi \left(0.4\right)$, ${\hat{H}}_{pha}\left({\hat{a}}_{3}\right)=2\pi \left(0.2\right)$ and ${\hat{H}}_{pha}\left({\hat{a}}_{4}\right)=2\pi \left(0.4\right)$.*

In Example 3, the approximate element of sub-parametric tuple ${\hat{℘}}_{1}$ (with 10% fuzzy parameterised grade) is

$$\left\{\begin{array}{c} \left({\hat{a}}_{1},\langle 0.2\;e^{j2\pi (0.3)},0.3\;e^{j2\pi (0.4)}\rangle \right),\left({\hat{a}}_{2},\langle 0.4\;e^{j2\pi (0.5)},0.5\;e^{j2\pi (0.6)}\rangle \right),\\ \left({\hat{a}}_{3},\langle 0.6\;e^{j2\pi (0.7)},0.3\;e^{j2\pi (0.8)}\rangle \right),\left({\hat{a}}_{4},\langle 0.8\;e^{j2\pi (0.4)},0.1\;e^{j2\pi (0.6)}\rangle \right) \end{array}\right\}$$

in which $\left({\hat{a}}_{1},\langle 0.2\;e^{j2\pi (0.3)},0.3\;e^{j2\pi (0.4)}\rangle \right)$ means that truth- and falsity-based amplitude values of ${\hat{a}}_{1}$ are 0.2 and 0.3, respectively, similarly truth- and falsity-based phase values of ${\hat{a}}_{1}$ are 2π(0.3) and 2π(0.4), respectively, in the approximation of sub-parametric tuple ${\hat{℘}}_{1}$. Similarly, all other terms can also be interpreted.

**Definition 6.**  *Let ${\hat{\Theta }}_{1}^{\hat{\Lambda }}$ and ${\hat{\Theta }}_{2}^{\hat{\Lambda }}$ be FPCIFHSSs over $\hat{\Lambda }$ such that*

(8) $${\hat{\Theta }}_{1}^{\hat{\Lambda }}=\left\{\left(\frac{{\hat{℘}}_{k}}{{\hat{\psi }}_{1}\left({\hat{℘}}_{k}\right)},\Psi_{1}\left(\frac{{\hat{℘}}_{k}}{{\hat{\psi }}_{1}\left({\hat{℘}}_{k}\right)}\right)\right)|\;{\hat{a}}_{i}\in \hat{\Lambda },\frac{{\hat{℘}}_{k}}{{\hat{\psi }}_{1}\left({\hat{℘}}_{k}\right)}\in {\hat{F}}_{1}\right\}$$

*where $\Psi_{1}\left(\frac{{\hat{℘}}_{k}}{{\hat{\psi }}_{1}\left({\hat{℘}}_{k}\right)}\right)=\langle {\hat{T}}_{\hat{\Theta }}^{1}\left(\frac{{\hat{℘}}_{k}}{{\hat{\psi }}_{1}\left({\hat{℘}}_{k}\right)}\right)\left({\hat{a}}_{i}\right),{\hat{F}}_{\hat{\Theta }}^{1}\left(\frac{{\hat{℘}}_{k}}{{\hat{\psi }}_{1}\left({\hat{℘}}_{k}\right)}\right)\left({\hat{a}}_{i}\right)\rangle$ with*

(9) $${\hat{T}}_{\hat{\Theta }}^{1}\left(\frac{{\hat{℘}}_{k}}{{\hat{\psi }}_{1}\left({\hat{℘}}_{k}\right)}\right)\left({\hat{a}}_{i}\right)={\hat{A}}_{\hat{T}}^{1}\left(\frac{{\hat{℘}}_{k}}{{\hat{\psi }}_{1}\left({\hat{℘}}_{k}\right)}\right)\left({\hat{a}}_{i}\right)\;e^{j{\hat{B}}_{\hat{T}}^{1}\left(\frac{{\hat{℘}}_{k}}{{\hat{\psi }}_{1}\left({\hat{℘}}_{k}\right)}\right)\left({\hat{a}}_{i}\right)}$$

(10) $${\hat{F}}_{\hat{\Theta }}^{1}\left(\frac{{\hat{℘}}_{k}}{{\hat{\psi }}_{1}\left({\hat{℘}}_{k}\right)}\right)\left({\hat{a}}_{i}\right)={\hat{A}}_{\hat{F}}^{1}\left(\frac{{\hat{℘}}_{k}}{{\hat{\psi }}_{1}\left({\hat{℘}}_{k}\right)}\right)\left({\hat{a}}_{i}\right)\;e^{j{\hat{B}}_{\hat{F}}^{1}\left(\frac{{\hat{℘}}_{k}}{{\hat{\psi }}_{1}\left({\hat{℘}}_{k}\right)}\right)\left({\hat{a}}_{i}\right)}$$

*and*

(11) $${\hat{\Theta }}_{2}^{\hat{\Lambda }}=\left\{\left(\frac{{\hat{℘}}_{k}}{{\hat{\psi }}_{2}\left({\hat{℘}}_{k}\right)},\Psi_{2}\left(\frac{{\hat{℘}}_{k}}{{\hat{\psi }}_{2}\left({\hat{℘}}_{k}\right)}\right)\right)|\;{\hat{a}}_{i}\in \hat{\Lambda },\frac{{\hat{℘}}_{k}}{{\hat{\psi }}_{2}\left({\hat{℘}}_{k}\right)}\in {\hat{F}}_{2}\right\}$$

*where $\Psi_{2}\left(\frac{{\hat{℘}}_{k}}{{\hat{\psi }}_{2}\left({\hat{℘}}_{k}\right)}\right)=\langle {\hat{T}}_{\hat{\Theta }}^{2}\left(\frac{{\hat{℘}}_{k}}{{\hat{\psi }}_{2}\left({\hat{℘}}_{k}\right)}\right)\left({\hat{a}}_{i}\right),{\hat{F}}_{\hat{\Theta }}^{2}\left(\frac{{\hat{℘}}_{k}}{{\hat{\psi }}_{2}\left({\hat{℘}}_{k}\right)}\right)\left({\hat{a}}_{i}\right)\rangle$ with*

(12) $${\hat{T}}_{\hat{\Theta }}^{2}\left(\frac{{\hat{℘}}_{k}}{{\hat{\psi }}_{2}\left({\hat{℘}}_{k}\right)}\right)\left({\hat{a}}_{i}\right)={\hat{A}}_{\hat{T}}^{2}\left(\frac{{\hat{℘}}_{k}}{{\hat{\psi }}_{2}\left({\hat{℘}}_{k}\right)}\right)\left({\hat{a}}_{i}\right)\;e^{j{\hat{B}}_{\hat{T}}^{2}\left(\frac{{\hat{℘}}_{k}}{{\hat{\psi }}_{2}\left({\hat{℘}}_{k}\right)}\right)\left({\hat{a}}_{i}\right)}$$

(13) $${\hat{F}}_{\hat{\Theta }}^{2}\left(\frac{{\hat{℘}}_{k}}{{\hat{\psi }}_{2}\left({\hat{℘}}_{k}\right)}\right)\left({\hat{a}}_{i}\right)={\hat{A}}_{\hat{F}}^{2}\left(\frac{{\hat{℘}}_{k}}{{\hat{\psi }}_{2}\left({\hat{℘}}_{k}\right)}\right)\left({\hat{a}}_{i}\right)\;e^{j{\hat{B}}_{\hat{F}}^{2}\left(\frac{{\hat{℘}}_{k}}{{\hat{\psi }}_{2}\left({\hat{℘}}_{k}\right)}\right)\left({\hat{a}}_{i}\right)}$$

*then*

*(1) ${\hat{\Theta }}_{1}^{\hat{\Lambda }}\cup {\hat{\Theta }}_{2}^{\hat{\Lambda }}={\hat{\Theta }}_{3}^{\hat{\Lambda }}$ is another FPCIFHSS over $\hat{\Lambda }$ such that*

$$\Psi_{3}\left(\frac{{\hat{℘}}_{k}}{{\hat{\psi }}_{3}\left({\hat{℘}}_{k}\right)}\right)=\left\{\begin{array}{cc} \begin{array}{c} \Psi_{1}\left(\frac{{\hat{℘}}_{k}}{{\hat{\psi }}_{1}\left({\hat{℘}}_{k}\right)}\right)\\ \Psi_{2}\left(\frac{{\hat{℘}}_{k}}{{\hat{\psi }}_{2}\left({\hat{℘}}_{k}\right)}\right)\\ \Psi_{3}\left(\frac{{\hat{℘}}_{k}}{{\hat{\psi }}_{3}^{{}^{\prime }}\left({\hat{℘}}_{k}\right)}\right) \end{array} & \begin{array}{c} {\hat{℘}}_{k}\in {\hat{ℑ}}_{1}\setminus {\hat{ℑ}}_{2}\\ {\hat{℘}}_{k}\in {\hat{ℑ}}_{2}\setminus {\hat{ℑ}}_{1}\\ {\hat{℘}}_{k}\in {\hat{ℑ}}_{1}\cap {\hat{ℑ}}_{2} \end{array} \end{array}\right.$$

*where $\Psi_{3}\left(\frac{{\hat{℘}}_{k}}{{\hat{\psi }}_{3}^{{}^{\prime }}\left({\hat{℘}}_{k}\right)}\right)=\Psi_{1}\left(\frac{{\hat{℘}}_{k}}{{\hat{\psi }}_{1}\left({\hat{℘}}_{k}\right)}\right)\cup \Psi_{2}\left(\frac{{\hat{℘}}_{k}}{{\hat{\psi }}_{2}\left({\hat{℘}}_{k}\right)}\right)$ with*

$${\hat{A}}_{\hat{T}}^{3}\left(\frac{{\hat{℘}}_{k}}{{\hat{\psi }}_{3}\left({\hat{℘}}_{k}\right)}\right)\left({\hat{a}}_{i}\right)=\max\left\{{\hat{A}}_{\hat{T}}^{1}\left(\frac{{\hat{℘}}_{k}}{{\hat{\psi }}_{1}\left({\hat{℘}}_{k}\right)}\right)\left({\hat{a}}_{i}\right),{\hat{A}}_{\hat{T}}^{2}\left(\frac{{\hat{℘}}_{k}}{{\hat{\psi }}_{2}\left({\hat{℘}}_{k}\right)}\right)\left({\hat{a}}_{i}\right)\right\},$$

$${\hat{A}}_{\hat{F}}^{3}\left(\frac{{\hat{℘}}_{k}}{{\hat{\psi }}_{3}\left({\hat{℘}}_{k}\right)}\right)\left({\hat{a}}_{i}\right)=\min\left\{{\hat{A}}_{\hat{F}}^{1}\left(\frac{{\hat{℘}}_{k}}{{\hat{\psi }}_{1}\left({\hat{℘}}_{k}\right)}\right)\left({\hat{a}}_{i}\right),{\hat{A}}_{\hat{F}}^{2}\left(\frac{{\hat{℘}}_{k}}{{\hat{\psi }}_{2}\left({\hat{℘}}_{k}\right)}\right)\left({\hat{a}}_{i}\right)\right\},$$

$${\hat{B}}_{\hat{T}}^{3}\left(\frac{{\hat{℘}}_{k}}{{\hat{\psi }}_{3}\left({\hat{℘}}_{k}\right)}\right)\left({\hat{a}}_{i}\right)=\max\left\{{\hat{B}}_{\hat{T}}^{1}\left(\frac{{\hat{℘}}_{k}}{{\hat{\psi }}_{1}\left({\hat{℘}}_{k}\right)}\right)\left({\hat{a}}_{i}\right),{\hat{B}}_{\hat{T}}^{2}\left(\frac{{\hat{℘}}_{k}}{{\hat{\psi }}_{2}\left({\hat{℘}}_{k}\right)}\right)\left({\hat{a}}_{i}\right)\right\},$$

$${\hat{B}}_{\hat{F}}^{3}\left(\frac{{\hat{℘}}_{k}}{{\hat{\psi }}_{3}\left({\hat{℘}}_{k}\right)}\right)\left({\hat{a}}_{i}\right)=\max\left\{{\hat{B}}_{\hat{F}}^{1}\left(\frac{{\hat{℘}}_{k}}{{\hat{\psi }}_{1}\left({\hat{℘}}_{k}\right)}\right)\left({\hat{a}}_{i}\right),{\hat{B}}_{\hat{F}}^{2}\left(\frac{{\hat{℘}}_{k}}{{\hat{\psi }}_{2}\left({\hat{℘}}_{k}\right)}\right)\left({\hat{a}}_{i}\right)\right\}$$

*and*

$${\hat{\psi }}_{3}\left({\hat{℘}}_{k}\right)=max\left\{{\hat{\psi }}_{1}\left({\hat{℘}}_{k}\right),{\hat{\psi }}_{2}\left({\hat{℘}}_{k}\right)\right\}.$$

*(2) ${\hat{\Theta }}_{1}^{\hat{\Lambda }}\cap {\hat{\Theta }}_{2}^{\hat{\Lambda }}={\hat{\Theta }}_{4}^{\hat{\Lambda }}$ is another FPCIFHSS over $\hat{\Lambda }$ such that*

$$\Psi_{4}\left(\frac{{\hat{℘}}_{k}}{{\hat{\psi }}_{4}\left({\hat{℘}}_{k}\right)}\right)=\Psi_{1}\left(\frac{{\hat{℘}}_{k}}{{\hat{\psi }}_{1}\left({\hat{℘}}_{k}\right)}\right)\cap \Psi_{2}\left(\frac{{\hat{℘}}_{k}}{{\hat{\psi }}_{2}\left({\hat{℘}}_{k}\right)}\right)$$

*with*

$${\hat{A}}_{\hat{T}}^{3}\left(\frac{{\hat{℘}}_{k}}{{\hat{\psi }}_{3}\left({\hat{℘}}_{k}\right)}\right)\left({\hat{a}}_{i}\right)=\min\left\{{\hat{A}}_{\hat{T}}^{1}\left(\frac{{\hat{℘}}_{k}}{{\hat{\psi }}_{1}\left({\hat{℘}}_{k}\right)}\right)\left({\hat{a}}_{i}\right),{\hat{A}}_{\hat{T}}^{2}\left(\frac{{\hat{℘}}_{k}}{{\hat{\psi }}_{2}\left({\hat{℘}}_{k}\right)}\right)\left({\hat{a}}_{i}\right)\right\},$$

$${\hat{A}}_{\hat{F}}^{3}\left(\frac{{\hat{℘}}_{k}}{{\hat{\psi }}_{3}\left({\hat{℘}}_{k}\right)}\right)\left({\hat{a}}_{i}\right)=\max\left\{{\hat{A}}_{\hat{F}}^{1}\left(\frac{{\hat{℘}}_{k}}{{\hat{\psi }}_{1}\left({\hat{℘}}_{k}\right)}\right)\left({\hat{a}}_{i}\right),{\hat{A}}_{\hat{F}}^{2}\left(\frac{{\hat{℘}}_{k}}{{\hat{\psi }}_{2}\left({\hat{℘}}_{k}\right)}\right)\left({\hat{a}}_{i}\right)\right\},$$

$${\hat{B}}_{\hat{T}}^{3}\left(\frac{{\hat{℘}}_{k}}{{\hat{\psi }}_{4}\left({\hat{℘}}_{k}\right)}\right)\left({\hat{a}}_{i}\right)=\min\left\{{\hat{B}}_{\hat{T}}^{1}\left(\frac{{\hat{℘}}_{k}}{{\hat{\psi }}_{1}\left({\hat{℘}}_{k}\right)}\right)\left({\hat{a}}_{i}\right),{\hat{B}}_{\hat{T}}^{2}\left(\frac{{\hat{℘}}_{k}}{{\hat{\psi }}_{2}\left({\hat{℘}}_{k}\right)}\right)\left({\hat{a}}_{i}\right)\right\},$$

$${\hat{B}}_{\hat{F}}^{3}\left(\frac{{\hat{℘}}_{k}}{{\hat{\psi }}_{4}\left({\hat{℘}}_{k}\right)}\right)\left({\hat{a}}_{i}\right)=\min\left\{{\hat{B}}_{\hat{F}}^{1}\left(\frac{{\hat{℘}}_{k}}{{\hat{\psi }}_{1}\left({\hat{℘}}_{k}\right)}\right)\left({\hat{a}}_{i}\right),{\hat{B}}_{\hat{F}}^{2}\left(\frac{{\hat{℘}}_{k}}{{\hat{\psi }}_{2}\left({\hat{℘}}_{k}\right)}\right)\left({\hat{a}}_{i}\right)\right\},$$

*and*

$${\hat{\psi }}_{4}\left({\hat{℘}}_{k}\right)=min\left\{{\hat{\psi }}_{1}\left({\hat{℘}}_{k}\right),{\hat{\psi }}_{2}\left({\hat{℘}}_{k}\right)\right\}.$$

**Example 4.**  *Reassuming Example 3, we have ${\hat{\Theta }}_{1}^{\hat{\Lambda }}=$*

$$\left\{\begin{array}{c} \left(\frac{{\hat{℘}}_{1}}{0.11},\left\{\begin{array}{c} \left({\hat{a}}_{1},\langle 0.21\;e^{j2\pi (0.32)},0.31\;e^{j2\pi (0.42)}\rangle \right),\left({\hat{a}}_{2},\langle 0.41\;e^{j2\pi (0.52)},0.51\;e^{j2\pi (0.62)}\rangle \right),\\ \left({\hat{a}}_{3},\langle 0.61\;e^{j2\pi (0.72)},0.31\;e^{j2\pi (0.82)}\rangle \right),\left({\hat{a}}_{4},\langle 0.81\;e^{j2\pi (0.42)},0.11\;e^{j2\pi (0.62)}\rangle \right) \end{array}\right\}\right),\\ \left(\frac{{\hat{℘}}_{2}}{0.31},\left\{\begin{array}{c} \left({\hat{a}}_{1},\langle 0.71\;e^{j2\pi (0.42)},0.11\;e^{j2\pi (0.32)}\rangle \right),\left({\hat{a}}_{2},\langle 0.61\;e^{j2\pi (0.52)},0.21\;e^{j2\pi (0.32)}\rangle \right),\\ \left({\hat{a}}_{3},\langle 0.51\;e^{j2\pi (0.62)},0.21\;e^{j2\pi (0.52)}\rangle \right),\left({\hat{a}}_{4},\langle 0.41\;e^{j2\pi (0.42)},0.31\;e^{j2\pi (0.52)}\rangle \right) \end{array}\right\}\right),\\ \left(\frac{{\hat{℘}}_{3}}{0.51},\left\{\begin{array}{c} \left({\hat{a}}_{1},\langle 0.81\;e^{j2\pi (0.62)},0.11\;e^{j2\pi (0.52)}\rangle \right),\left({\hat{a}}_{2},\langle 0.71\;e^{j2\pi (0.52)},0.21\;e^{j2\pi (0.42)}\rangle \right),\\ \left({\hat{a}}_{3},\langle 0.61\;e^{j2\pi (0.62)},0.11\;e^{j2\pi (0.12)}\rangle \right),\left({\hat{a}}_{4},\langle 0.51\;e^{j2\pi (0.32)},0.31\;e^{j2\pi (0.62)}\rangle \right) \end{array}\right\}\right),\\ \left(\frac{{\hat{℘}}_{4}}{0.61},\left\{\begin{array}{c} \left({\hat{a}}_{1},\langle 0.51\;e^{j2\pi (0.12)},0.11\;e^{j2\pi (0.12)}\rangle \right),\left({\hat{a}}_{2},\langle 0.41\;e^{j2\pi (0.22)},0.31\;e^{j2\pi (0.22)}\rangle \right),\\ \left({\hat{a}}_{3},\langle 0.31\;e^{j2\pi (0.12)},0.11\;e^{j2\pi (0.52)}\rangle \right),\left({\hat{a}}_{4},\langle 0.21\;e^{j2\pi (0.62)},0.41\;e^{j2\pi (0.62)}\rangle \right) \end{array}\right\}\right) \end{array}\right\}$$

*                            and ${\hat{\Theta }}_{2}^{\hat{\Lambda }}=$*

$$\left\{\begin{array}{c} \left(\frac{{\hat{℘}}_{1}}{0.12},\left\{\begin{array}{c} \left({\hat{a}}_{1},\langle 0.22\;e^{j2\pi (0.31)},0.32\;e^{j2\pi (0.41)}\rangle \right),\left({\hat{a}}_{2},\langle 0.42\;e^{j2\pi (0.51)},0.52\;e^{j2\pi (0.61)}\rangle \right),\\ \left({\hat{a}}_{3},\langle 0.62\;e^{j2\pi (0.71)},0.32\;e^{j2\pi (0.81)}\rangle \right),\left({\hat{a}}_{4},\langle 0.82\;e^{j2\pi (0.41)},0.12\;e^{j2\pi (0.61)}\rangle \right) \end{array}\right\}\right),\\ \left(\frac{{\hat{℘}}_{2}}{0.32},\left\{\begin{array}{c} \left({\hat{a}}_{1},\langle 0.72\;e^{j2\pi (0.41)},0.12\;e^{j2\pi (0.31)}\rangle \right),\left({\hat{a}}_{2},\langle 0.62\;e^{j2\pi (0.51)},0.22\;e^{j2\pi (0.31)}\rangle \right),\\ \left({\hat{a}}_{3},\langle 0.52\;e^{j2\pi (0.61)},0.22\;e^{j2\pi (0.51)}\rangle \right),\left({\hat{a}}_{4},\langle 0.42\;e^{j2\pi (0.41)},0.32\;e^{j2\pi (0.51)}\rangle \right) \end{array}\right\}\right),\\ \left(\frac{{\hat{℘}}_{3}}{0.52},\left\{\begin{array}{c} \left({\hat{a}}_{1},\langle 0.82\;e^{j2\pi (0.61)},0.12\;e^{j2\pi (0.51)}\rangle \right),\left({\hat{a}}_{2},\langle 0.72\;e^{j2\pi (0.51)},0.22\;e^{j2\pi (0.41)}\rangle \right),\\ \left({\hat{a}}_{3},\langle 0.62\;e^{j2\pi (0.61)},0.12\;e^{j2\pi (0.11)}\rangle \right),\left({\hat{a}}_{4},\langle 0.52\;e^{j2\pi (0.31)},0.32\;e^{j2\pi (0.61)}\rangle \right) \end{array}\right\}\right),\\ \left(\frac{{\hat{℘}}_{4}}{0.62},\left\{\begin{array}{c} \left({\hat{a}}_{1},\langle 0.52\;e^{j2\pi (0.11)},0.12\;e^{j2\pi (0.11)}\rangle \right),\left({\hat{a}}_{2},\langle 0.42\;e^{j2\pi (0.21)},0.32\;e^{j2\pi (0.21)}\rangle \right),\\ \left({\hat{a}}_{3},\langle 0.32\;e^{j2\pi (0.11)},0.12\;e^{j2\pi (0.51)}\rangle \right),\left({\hat{a}}_{4},\langle 0.22\;e^{j2\pi (0.61)},0.42\;e^{j2\pi (0.61)}\rangle \right) \end{array}\right\}\right) \end{array}\right\}$$

*                            then ${\hat{\Theta }}_{3}^{\hat{\Lambda }}={\hat{\Theta }}_{1}^{\hat{\Lambda }}\cup {\hat{\Theta }}_{2}^{\hat{\Lambda }}$*

$$\left\{\begin{array}{c} \left(\frac{{\hat{℘}}_{1}}{0.12},\left\{\begin{array}{c} \left({\hat{a}}_{1},\langle 0.22\;e^{j2\pi (0.32)},0.31\;e^{j2\pi (0.42)}\rangle \right),\left({\hat{a}}_{2},\langle 0.42\;e^{j2\pi (0.52)},0.51\;e^{j2\pi (0.62)}\rangle \right),\\ \left({\hat{a}}_{3},\langle 0.62\;e^{j2\pi (0.72)},0.31\;e^{j2\pi (0.82)}\rangle \right),\left({\hat{a}}_{4},\langle 0.82\;e^{j2\pi (0.42)},0.11\;e^{j2\pi (0.62)}\rangle \right) \end{array}\right\}\right),\\ \left(\frac{{\hat{℘}}_{2}}{0.32},\left\{\begin{array}{c} \left({\hat{a}}_{1},\langle 0.72\;e^{j2\pi (0.42)},0.11\;e^{j2\pi (0.32)}\rangle \right),\left({\hat{a}}_{2},\langle 0.62\;e^{j2\pi (0.52)},0.21\;e^{j2\pi (0.32)}\rangle \right),\\ \left({\hat{a}}_{3},\langle 0.52\;e^{j2\pi (0.62)},0.21\;e^{j2\pi (0.52)}\rangle \right),\left({\hat{a}}_{4},\langle 0.42\;e^{j2\pi (0.42)},0.31\;e^{j2\pi (0.52)}\rangle \right) \end{array}\right\}\right),\\ \left(\frac{{\hat{℘}}_{3}}{0.52},\left\{\begin{array}{c} \left({\hat{a}}_{1},\langle 0.82\;e^{j2\pi (0.62)},0.11\;e^{j2\pi (0.52)}\rangle \right),\left({\hat{a}}_{2},\langle 0.72\;e^{j2\pi (0.52)},0.21\;e^{j2\pi (0.42)}\rangle \right),\\ \left({\hat{a}}_{3},\langle 0.62\;e^{j2\pi (0.62)},0.11\;e^{j2\pi (0.12)}\rangle \right),\left({\hat{a}}_{4},\langle 0.52\;e^{j2\pi (0.32)},0.31\;e^{j2\pi (0.62)}\rangle \right) \end{array}\right\}\right),\\ \left(\frac{{\hat{℘}}_{4}}{0.62},\left\{\begin{array}{c} \left({\hat{a}}_{1},\langle 0.52\;e^{j2\pi (0.12)},0.11\;e^{j2\pi (0.12)}\rangle \right),\left({\hat{a}}_{2},\langle 0.42\;e^{j2\pi (0.22)},0.31\;e^{j2\pi (0.22)}\rangle \right),\\ \left({\hat{a}}_{3},\langle 0.32\;e^{j2\pi (0.12)},0.11\;e^{j2\pi (0.52)}\rangle \right),\left({\hat{a}}_{4},\langle 0.22\;e^{j2\pi (0.62)},0.41\;e^{j2\pi (0.62)}\rangle \right) \end{array}\right\}\right) \end{array}\right\}$$

*                            and ${\hat{\Theta }}_{4}^{\hat{\Lambda }}={\hat{\Theta }}_{1}^{\hat{\Lambda }}\cap {\hat{\Theta }}_{2}^{\hat{\Lambda }}$*

$$\left\{\begin{array}{c} \left(\frac{{\hat{℘}}_{1}}{0.11},\left\{\begin{array}{c} \left({\hat{a}}_{1},\langle 0.21\;e^{j2\pi (0.31)},0.32\;e^{j2\pi (0.41)}\rangle \right),\left({\hat{a}}_{2},\langle 0.41\;e^{j2\pi (0.51)},0.52\;e^{j2\pi (0.61)}\rangle \right),\\ \left({\hat{a}}_{3},\langle 0.61\;e^{j2\pi (0.71)},0.32\;e^{j2\pi (0.81)}\rangle \right),\left({\hat{a}}_{4},\langle 0.81\;e^{j2\pi (0.41)},0.12\;e^{j2\pi (0.61)}\rangle \right) \end{array}\right\}\right),\\ \left(\frac{{\hat{℘}}_{2}}{0.31},\left\{\begin{array}{c} \left({\hat{a}}_{1},\langle 0.71\;e^{j2\pi (0.41)},0.12\;e^{j2\pi (0.31)}\rangle \right),\left({\hat{a}}_{2},\langle 0.61\;e^{j2\pi (0.51)},0.22\;e^{j2\pi (0.31)}\rangle \right),\\ \left({\hat{a}}_{3},\langle 0.51\;e^{j2\pi (0.61)},0.22\;e^{j2\pi (0.51)}\rangle \right),\left({\hat{a}}_{4},\langle 0.41\;e^{j2\pi (0.41)},0.32\;e^{j2\pi (0.51)}\rangle \right) \end{array}\right\}\right),\\ \left(\frac{{\hat{℘}}_{3}}{0.51},\left\{\begin{array}{c} \left({\hat{a}}_{1},\langle 0.81\;e^{j2\pi (0.61)},0.12\;e^{j2\pi (0.51)}\rangle \right),\left({\hat{a}}_{2},\langle 0.71\;e^{j2\pi (0.51)},0.22\;e^{j2\pi (0.41)}\rangle \right),\\ \left({\hat{a}}_{3},\langle 0.61\;e^{j2\pi (0.61)},0.12\;e^{j2\pi (0.11)}\rangle \right),\left({\hat{a}}_{4},\langle 0.51\;e^{j2\pi (0.31)},0.32\;e^{j2\pi (0.61)}\rangle \right) \end{array}\right\}\right),\\ \left(\frac{{\hat{℘}}_{4}}{0.61},\left\{\begin{array}{c} \left({\hat{a}}_{1},\langle 0.51\;e^{j2\pi (0.11)},0.12\;e^{j2\pi (0.11)}\rangle \right),\left({\hat{a}}_{2},\langle 0.41\;e^{j2\pi (0.21)},0.32\;e^{j2\pi (0.21)}\rangle \right),\\ \left({\hat{a}}_{3},\langle 0.31\;e^{j2\pi (0.11)},0.12\;e^{j2\pi (0.51)}\rangle \right),\left({\hat{a}}_{4},\langle 0.21\;e^{j2\pi (0.61)},0.42\;e^{j2\pi (0.61)}\rangle \right) \end{array}\right\}\right) \end{array}\right\}.$$

### 3.2. Role of Fuzzy Parameterisation in Analysis of Susceptibility to Brain Tumours

The idea of fuzzy parameterisation is meant to tackle uncertainties attached to the procedure of selecting parameters for the analysis and ranking of objects under observation. Let $\hat{\Lambda }=\left\{{\hat{a}}_{1},{\hat{a}}_{2},{\hat{a}}_{3},\ldots ,{\hat{a}}_{\alpha }\right\}$ be a space of objects under consideration and $\hat{F}=\left\{{\hat{℘}}_{1}/\hat{\psi }\left({\hat{℘}}_{1}\right),{\hat{℘}}_{2}/\hat{\psi }\left({\hat{℘}}_{2}\right),\ldots ,{\hat{℘}}_{r}/\hat{\psi }\left({\hat{℘}}_{\beta }\right)\right\}$ be an FS over the set of attribute-valued tuples $\hat{ℑ}=\left\{{\hat{℘}}_{1},{\hat{℘}}_{2},\ldots ,{\hat{℘}}_{\beta }\right\}$. Let ${\hat{A}}_{\hat{T}}^{i}\left({\hat{℘}}_{1}/\hat{\psi }\left({\hat{℘}}_{1}\right)\right)$ and ${\hat{A}}_{\hat{F}}^{i}\left({\hat{℘}}_{1}/\hat{\psi }\left({\hat{℘}}_{1}\right)\right)$ be amplitude values of ${\hat{a}}_{i},i=1,2,\ldots ,\alpha$ in true-belonging and false-belonging components of CIFNs with respect to ${\hat{℘}}_{1}/\hat{\psi }\left({\hat{℘}}_{1}\right)$. Similarly, let ${\hat{B}}_{\hat{T}}^{i}\left({\hat{℘}}_{1}/\hat{\psi }\left({\hat{℘}}_{1}\right)\right)$ and ${\hat{B}}_{\hat{F}}^{i}\left({\hat{℘}}_{1}/\hat{\psi }\left({\hat{℘}}_{1}\right)\right)$ be phase values of ${\hat{a}}_{i}$ in true-belonging and false-belonging components of CIFNs with respect to ${\hat{℘}}_{1}/\hat{\psi }\left({\hat{℘}}_{1}\right)$. Then, the fuzzy parameterised value $\hat{\psi }\left({\hat{℘}}_{1}\right)$ of ${\hat{℘}}_{1}$ can be computed as

(14) $$\hat{\psi }\left({\hat{℘}}_{1}\right)=\frac{1}{2}\left\{\begin{array}{c} \frac{max\left\{{\hat{A}}_{\hat{T}}^{i}\left({\hat{℘}}_{1}\right)\right\}+min\left\{{\hat{A}}_{\hat{F}}^{i}\left({\hat{℘}}_{1}\right)\right\}}{2}+\frac{max\left\{{\hat{B}}_{\hat{T}}^{i}\left({\hat{℘}}_{1}\right)\right\}+min\left\{{\hat{B}}_{\hat{F}}^{i}\left({\hat{℘}}_{1}\right)\right\}}{2} \end{array}\right\}.$$

### 3.3. Algebraic Criterion for the Transformation of CIFNs

If $\langle {\hat{A}}_{\hat{T}}\left({\hat{a}}_{i}\right)\;e^{j{\hat{B}}_{\hat{T}}\left({\hat{a}}_{i}\right)},{\hat{A}}_{\hat{F}}\left({\hat{a}}_{i}\right)\;e^{j{\hat{B}}_{\hat{F}}\left({\hat{a}}_{i}\right)}\rangle$ is a CIFN for ${\hat{a}}_{i}$$\in \hat{\Lambda }$ corresponding to fuzzy parameterised tuples $\frac{{\hat{℘}}_{k}}{\hat{\psi }\left({\hat{℘}}_{k}\right)}\in \hat{F}$, then CIFN can be converted to CFN by using the following arithmetical formula

(15) $${\hat{\nabla }}_{CFN}=\langle \frac{|{\hat{A}}_{\hat{T}}\left({\hat{a}}_{i}\right)-{\hat{A}}_{\hat{F}}\left({\hat{a}}_{i}\right)|}{2},\frac{{\hat{B}}_{\hat{T}}\left({\hat{a}}_{i}\right)+{\hat{B}}_{\hat{F}}\left({\hat{a}}_{i}\right)}{4\pi }\rangle .$$

If ${\hat{\nabla }}_{CFN}=\langle {\hat{\varpi }}_{1},{\hat{\varpi }}_{2}\rangle$ is a CFN, then fuzzy values can be obtained from it by using the following formula

(16) $$A_{FN}=\frac{|{\hat{\varpi }}_{1}-{\hat{\varpi }}_{2}|}{2}.$$

### 3.4. Selection Criterion for Parameters and Sub-Parameters

The characteristics (criteria) and sub-attributes (sub-criteria) are the main elements that directly connect to the MADM problem and potentially have a significant impact on decisions. Because of this, it is advised to choose parameters and sub-parameters with intelligence. Interviewing people and conducting questionnaire-based surveys are seen as appropriate methods for gathering information that will help select parameters and sub-parameters. However, only the parameters and sub-parameters compatible with the chosen algebraic model will likely be taken into account. Only those parameters (criteria) that are likely to be divided into disjoint sub-classes with sub-parametric values (sub-criteria) are accepted after reviewing the pertinent literature and utilising the suggested model.

**Figure 2:**
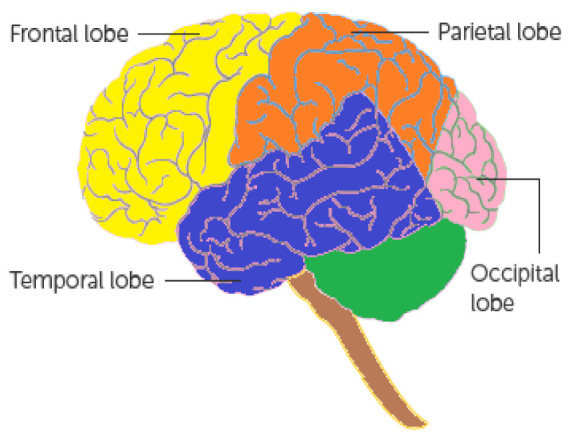
Classification of cerebrum (source: [39]).

**Figure 3:**
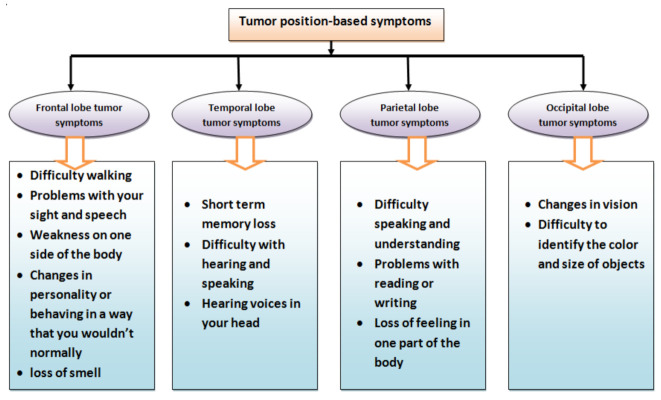
Classification of tumour location-based symptoms (source: [39]).

According to the American Association of Neurological Surgeons [38], many types of tumours may appear in the body for several uncertain reasons, but this study considered only those tumours which relate to the brain based on their positions in the cerebrum. A brief description of them is provided in Figure 2. Since there are many symptoms which may lead to the suffering of brain tumours [39,40], the most relevant symptoms and their sub-categories are considered as parameters (criteria) and sub-parameters (sub-criteria) for the evaluation of susceptibility to brain tumours. The purpose of choosing them is based on their relevance and suitability with respect to brain tumours. Figure 3 presents a brief description of adopted modified parameters and sub-parameters. To study their roles in detail, one can visit the web pages [39,40].

### 3.5. Profile of Decision Makers and Their Roles

The key players in MADM practice are decision makers (experts) who make the decisions necessary for the evaluation process to be completed successfully. Their shared conflicts of interest or other relevant disagreements might result in biased judgments. Therefore, it is common to employ professionals with multi-disciplinary competence areas from many sources (departments). A few of the main duties of decision makers in MADM include the following:

1. Analysing raw data (information) gathered from various sources.
2. Processing the analysed data statistically.
3. Examination of options and constraints.
4. Analysing processed data using various parameters.
5. A concise set of appropriate parameters for assessing alternatives.
6. Using an appropriate algebraic model to offer recommendations for approximating options based on parameters.
7. Sorting of objects under observation.

In the present study, the decision makers are qualified doctors such as neurologists and neuropathologists who specialise in issues concerning the brain and central nervous system. Although they can manage a major part of the evaluation easily, due to the involvement of many other factors, they can be assisted by other doctors such as internists, oncologists, radiation oncologists, neuro-oncologists and neurosurgeons [40].

### 3.6. Designing of Decision Support System

In this section, a decision support mechanism is presented, which assists the decision makers in assessing the susceptibility of patients to brain tumours by following the easy steps of the proposed robust algorithm.

Patients with brain tumours are typically directed to the oncology department at urban hospitals in some developing Asian countries such as Pakistan. There, the tumours’ conditions are first assessed before an appropriate course of therapy is suggested. Lack of resources makes it nearly impossible to diagnose a particular type of brain tumour and then treat it locally. Therefore, it is preferable to just evaluate a patient’s susceptibility to brain tumours at this local level before referring them to the appropriate facilities for treatment.

Now, a robust algorithm is being proposed using the aggregations of the proposed algebraic model FPCIFHSS and other arithmetical cum decision-making techniques.

**Figure 4:**
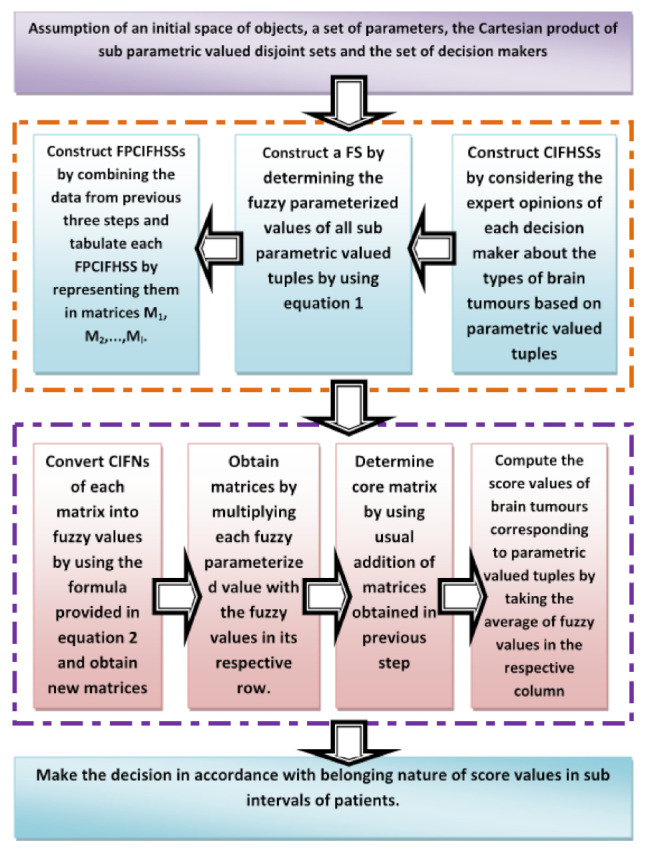
Flowchart of proposed PMBSA algorithm.

A brief step-wise description of Algorithm 1 is presented in Figure 4. Now, Algorithm 1 is explained by the following case study-based numerical example. In this case, the opinions of decision makers are hypothetical.
**Algorithm 1 Pythagorean Means based Scoring Algorithm (PMBSA):** The algorithm is divided into following four major stages**Input:**(1).Assume the sets like $\hat{\Lambda }=\left\{{\hat{a}}_{1},{\hat{a}}_{2},{\hat{a}}_{3},\ldots ,{\hat{a}}_{n}\right\}$ as initial spaces of objects consisting of various types of tumours, $\hat{\Xi }=\left\{{\hat{e}}_{1},{\hat{e}}_{2},{\hat{e}}_{3},\ldots ,{\hat{e}}_{m}\right\}$ as a set of parameters consisting of relevant symptoms of brain tumours, $\hat{ℑ}=\left\{{\hat{℘}}_{1},{\hat{℘}}_{2},{\hat{℘}}_{3},\ldots ,{\hat{℘}}_{k}\right\}$ as Cartesian product of ${\hat{\Xi }}_{i},i\in \left\{1,2,3,\ldots ,m\right\}$ where ${\hat{\Xi }}_{i}$ are non-overlapping sets consisting of sub-parametric values of ${\hat{e}}_{i}\in \hat{\Xi }$ and $\hat{X}=\left\{{Dm}_{1},{Dm}_{2},{Dm}_{3},\ldots ,{Dm}_{l}\right\}$ as a set of decision makers consisting of some neurologists and neuropathologists.**Construction:**(2).Construct CIFHSSs by considering the expert opinions of each decision maker about the types of brain tumours based on parametric valued tuples of $\hat{ℑ}$.(3).Construct an FS $\hat{F}$ over $\hat{ℑ}$ by determining the fuzzy parameterised values of all ${\hat{℘}}_{r},r\in \left\{1,2,3,\ldots ,k\right\}$ in accordance with Equation (14).(4).Construct FPCIFHSSs by combining the data from the previous three steps and tabulate each FPCIFHSS by representing them in matrices $M_{1},M_{2},\ldots ,M_{l}$.**Computation:**(5).Convert CIFNs of each matrix into fuzzy values by using the formula provided in Equation (15) and obtain new matrices $M_{1}^{fpf},M_{2}^{fpf},\ldots ,M_{l}^{fpf}$.(6).Obtain matrices $M_{1}^{f},M_{2}^{f},\ldots ,M_{l}^{f}$ by multiplying each fuzzy parameterised value with the fuzzy values in its respective row.(7).Determine core matrix $M^{core}$ by using algebraic formula $M^{core}=M_{1}^{f}⊕M_{2}^{f}⊕\ldots ⊕M_{l}^{f}$ where ⊕ is meant for the usual addition of matrices.(8).Compute the score values $\hat{\partial }\left({\hat{a}}_{i}\right),i=1,2,3,\ldots ,n$ of brain tumours corresponding to parametric valued tuples ${\hat{℘}}_{j},j=1,2,3,\ldots ,k$ by taking the average of fuzzy values appearing in the respective column of ${\hat{a}}_{i}$.**Output:**(9).Make the decision in accordance with the belonging nature of score values in sub-intervals of patients.

**Example 5.**  *The administration of a cancer hospital, “MEDICARE" (a hypothetical name), is very much concerned with the increasing ratio of suspected brain tumour patients being referred by various hospitals from all over the country. In order to evaluate the susceptibility level of patients to the specific type of brain tumours, a departmental committee is constituted consisting of two neurologists and one neuropathologist, which is considered as a set of decision makers $\hat{X}=\left\{{Dm}_{1},{Dm}_{2},{Dm}_{3}\right\}$. According to the terms and conditions provided to the committee, the following are the tasks of the committee:*

*1. Shortlist the patients for analysis of susceptibility to brain tumours and assign susceptibility degrees as subintervals of [0, 1].*

*2. List the expected types of brain tumours and their related symptoms after a close analysis of the literature and other related sources.*

*3. Provide opinions for the approximations of brain tumour types separately based on multi-argument-based symptoms by considering fuzzy parameterisation, complex intuitionistic fuzzy setting and hypersoft setting.*

**Figure 5:**
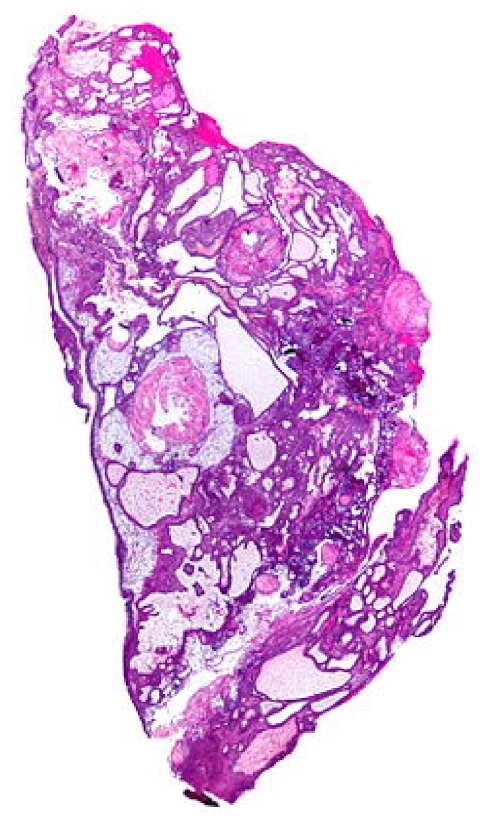
Craniopharyngioma: brain tumour of type 1 (source: https://en.wikipedia.org/wiki/Craniopharyngioma) (accessed on 16 October 2022).

**Figure 6:**
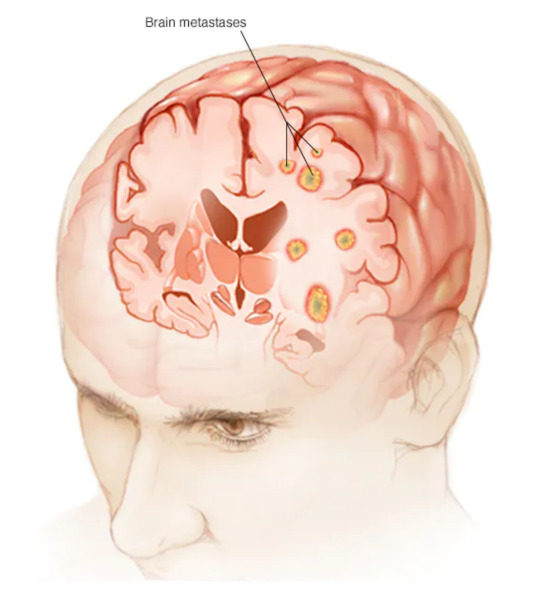
Brain-metastases: brain tumour of type 2 (source: https://www.mayoclinic.org/diseases-conditions/brain-metastases/symptoms-causes/syc-20350136) (accessed on 18 September 2022).

**Figure 7:**
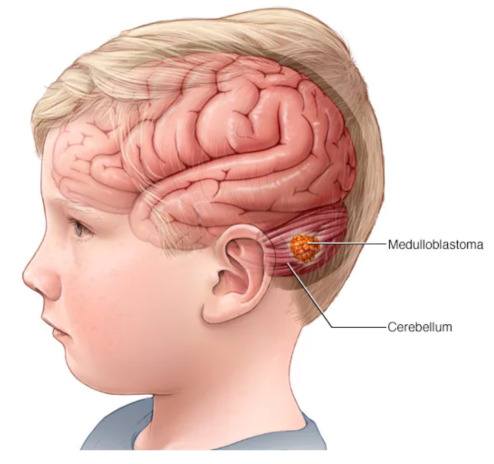
Medulloblastomas: brain tumour of type 3 (source: https://www.mayoclinic.org/diseases-conditions/medulloblastoma/cdc-20363524) (accessed on 18 September 2022).

*After mutual consultation, three types of brain tumours are shortlisted which are enclosed as a set of initial spaces of objects $\hat{\Lambda }=\left\{{\hat{a}}_{1}=craniopharyngioma,{\hat{a}}_{2}=brain-metastases,{\hat{a}}_{3}=medulloblastomas\right\}$. Figure 5, Figure 6 and Figure 7 present the pictorial display of these types of tumours, respectively. Six patients, ${\hat{P}}_{1}$, ${\hat{P}}_{2}$, ${\hat{P}}_{3}$, ${\hat{P}}_{4}$, ${\hat{P}}_{5}$ and ${\hat{P}}_{6}$, are shortlisted and their susceptibility degrees are supposed to be contained in subintervals [0, 0.3), [0.2, 0.4), [0.2, 0.6), [0.3, 0.7), [0.3, 0.85) and [0.85, 1], respectively. The committee has selected some appropriate symptoms in accordance with the location of brain tumours and considered them as evaluating parameters for this case. These symptoms form a set of parameters $\hat{\Xi }=\left\{\begin{array}{c} {\hat{e}}_{1}=frontal\;lobe\;tumour\;symptoms,{\hat{e}}_{2}=temporal\;lobe\;tumour\;symptoms,\\ {\hat{e}}_{3}=parietal\;lobe\;tumour\;symptoms,{\hat{e}}_{4}=occipetal\;lobe\;tumour\;symptoms \end{array}\right\}$. For the sake of having reliable evaluations, the chosen parameters are then classified on a preferential basis into their respective sub-parametric valued non-overlapping sets:*

*${\hat{\Xi }}_{1}=\left\{\begin{array}{c} {\hat{e}}_{11}=problems\;with\;sight\;and\;speech,{\hat{e}}_{12}=loss\;of\;smell \end{array}\right\}$,*

*${\hat{\Xi }}_{2}=\left\{\begin{array}{c} {\hat{e}}_{21}=short\;term\;memory\;loss,{\hat{e}}_{22}=hearing\;voices\;in\;head \end{array}\right\}$,*

*${\hat{\Xi }}_{3}=\left\{\begin{array}{c} {\hat{e}}_{31}=loss\;of\;feeling\;in\;one\;part\;of\;the\;body \end{array}\right\}$,*

*${\hat{\Xi }}_{4}=\left\{\begin{array}{c} {\hat{e}}_{41}=difficulty\;to\;identify\;the\;colour\;and\;size\;of\;objects \end{array}\right\}$.*

*For the sake of including multiple arguments simultaneously, the Cartesian product of ${\hat{\Xi }}_{i},i=1,2,3,4$ is calculated, that is*

$\hat{\Xi }={\hat{\Xi }}_{1}\times {\hat{\Xi }}_{2}\times {\hat{\Xi }}_{3}\times {\hat{\Xi }}_{4}$

*$\hat{\Xi }=\left\{\begin{array}{c} {\hat{℘}}_{1}=\left({\hat{e}}_{11},{\hat{e}}_{21},{\hat{e}}_{31},{\hat{e}}_{41}\right),{\hat{℘}}_{2}=\left({\hat{e}}_{11},{\hat{e}}_{22},{\hat{e}}_{31},{\hat{e}}_{41}\right),\\ {\hat{℘}}_{3}=\left({\hat{e}}_{12},{\hat{e}}_{21},{\hat{e}}_{31},{\hat{e}}_{41}\right),{\hat{℘}}_{4}=\left({\hat{e}}_{12},{\hat{e}}_{22},{\hat{e}}_{31},{\hat{e}}_{41}\right) \end{array}\right\}$.*

*Now, all the members provide their expert opinions separately for the approximations of three types of brain tumours based on sub-parametric valued tuples ${\hat{℘}}_{1}$, ${\hat{℘}}_{2}$, ${\hat{℘}}_{3}$ and ${\hat{℘}}_{4}$ in terms of CIFNs, that is*

    *For ${Dm}_{1}$:*

$$\Psi \left({\hat{℘}}_{1}\right)=\left\{\begin{array}{c} \left({\hat{a}}_{1},\langle 0.12\;e^{j2\pi (0.13)},0.13\;e^{j2\pi (0.14)}\rangle \right),\\ \left({\hat{a}}_{2},\langle 0.14\;e^{j2\pi (0.15)},0.15\;e^{j2\pi (0.16)}\rangle \right),\\ \left({\hat{a}}_{3},\langle 0.16\;e^{j2\pi (0.17)},0.13\;e^{j2\pi (0.18)}\rangle \right) \end{array}\right\}.$$

$$\Psi \left({\hat{℘}}_{2}\right)=\left\{\begin{array}{c} \left({\hat{a}}_{1},\langle 0.22\;e^{j2\pi (0.23)},0.23\;e^{j2\pi (0.24)}\rangle \right),\\ \left({\hat{a}}_{2},\langle 0.24\;e^{j2\pi (0.25)},0.25\;e^{j2\pi (0.26)}\rangle \right),\\ \left({\hat{a}}_{3},\langle 0.26\;e^{j2\pi (0.27)},0.23\;e^{j2\pi (0.28)}\rangle \right) \end{array}\right\},$$

$$\Psi \left({\hat{℘}}_{3}\right)=\left\{\begin{array}{c} \left({\hat{a}}_{1},\langle 0.32\;e^{j2\pi (0.33)},0.33\;e^{j2\pi (0.34)}\rangle \right),\\ \left({\hat{a}}_{2},\langle 0.34\;e^{j2\pi (0.35)},0.35\;e^{j2\pi (0.36)}\rangle \right),\\ \left({\hat{a}}_{3},\langle 0.36\;e^{j2\pi (0.37)},0.33\;e^{j2\pi (0.38)}\rangle \right) \end{array}\right\},$$

$$\Psi \left({\hat{℘}}_{4}\right)=\left\{\begin{array}{c} \left({\hat{a}}_{1},\langle 0.42\;e^{j2\pi (0.43)},0.43\;e^{j2\pi (0.44)}\rangle \right),\\ \left({\hat{a}}_{2},\langle 0.44\;e^{j2\pi (0.45)},0.45\;e^{j2\pi (0.46)}\rangle \right),\\ \left({\hat{a}}_{3},\langle 0.46\;e^{j2\pi (0.47)},0.43\;e^{j2\pi (0.48)}\rangle \right) \end{array}\right\}.$$

    *For ${Dm}_{2}$:*

$$\Psi \left({\hat{℘}}_{1}\right)=\left\{\begin{array}{c} \left({\hat{a}}_{1},\langle 0.21\;e^{j2\pi (0.31)},0.31\;e^{j2\pi (0.41)}\rangle \right),\\ \left({\hat{a}}_{2},\langle 0.41\;e^{j2\pi (0.51)},0.51\;e^{j2\pi (0.61)}\rangle \right),\\ \left({\hat{a}}_{3},\langle 0.61\;e^{j2\pi (0.71)},0.31\;e^{j2\pi (0.81)}\rangle \right) \end{array}\right\},$$

$$\Psi \left({\hat{℘}}_{2}\right)=\left\{\begin{array}{c} \left({\hat{a}}_{1},\langle 0.22\;e^{j2\pi (0.32)},0.32\;e^{j2\pi (0.42)}\rangle \right),\\ \left({\hat{a}}_{2},\langle 0.42\;e^{j2\pi (0.52)},0.52\;e^{j2\pi (0.62)}\rangle \right),\\ \left({\hat{a}}_{3},\langle 0.62\;e^{j2\pi (0.72)},0.32\;e^{j2\pi (0.82)}\rangle \right) \end{array}\right\},$$

$$\Psi \left({\hat{℘}}_{3}\right)=\left\{\begin{array}{c} \left({\hat{a}}_{1},\langle 0.23\;e^{j2\pi (0.33)},0.33\;e^{j2\pi (0.43)}\rangle \right),\\ \left({\hat{a}}_{2},\langle 0.43\;e^{j2\pi (0.53)},0.53\;e^{j2\pi (0.63)}\rangle \right),\\ \left({\hat{a}}_{3},\langle 0.63\;e^{j2\pi (0.73)},0.33\;e^{j2\pi (0.83)}\rangle \right) \end{array}\right\},$$

$$\Psi \left({\hat{℘}}_{4}\right)=\left\{\begin{array}{c} \left({\hat{a}}_{1},\langle 0.24\;e^{j2\pi (0.34)},0.34\;e^{j2\pi (0.44)}\rangle \right),\\ \left({\hat{a}}_{2},\langle 0.44\;e^{j2\pi (0.54)},0.54\;e^{j2\pi (0.64)}\rangle \right),\\ \left({\hat{a}}_{3},\langle 0.64\;e^{j2\pi (0.74)},0.34\;e^{j2\pi (0.84)}\rangle \right) \end{array}\right\}.$$

    *For ${Dm}_{3}$:*

$$\Psi \left({\hat{℘}}_{1}\right)=\left\{\begin{array}{c} \left({\hat{a}}_{1},\langle 0.24\;e^{j2\pi (0.34)},0.34\;e^{j2\pi (0.44)}\rangle \right),\\ \left({\hat{a}}_{2},\langle 0.44\;e^{j2\pi (0.54)},0.54\;e^{j2\pi (0.64)}\rangle \right),\\ \left({\hat{a}}_{3},\langle 0.64\;e^{j2\pi (0.74)},0.34\;e^{j2\pi (0.84)}\rangle \right) \end{array}\right\},$$

$$\Psi \left({\hat{℘}}_{2}\right)=\left\{\begin{array}{c} \left({\hat{a}}_{1},\langle 0.23\;e^{j2\pi (0.33)},0.33\;e^{j2\pi (0.43)}\rangle \right),\\ \left({\hat{a}}_{2},\langle 0.43\;e^{j2\pi (0.53)},0.53\;e^{j2\pi (0.63)}\rangle \right),\\ \left({\hat{a}}_{3},\langle 0.63\;e^{j2\pi (0.73)},0.33\;e^{j2\pi (0.83)}\rangle \right) \end{array}\right\},$$

$$\Psi \left({\hat{℘}}_{3}\right)=\left\{\begin{array}{c} \left({\hat{a}}_{1},\langle 0.22\;e^{j2\pi (0.32)},0.32\;e^{j2\pi (0.42)}\rangle \right),\\ \left({\hat{a}}_{2},\langle 0.42\;e^{j2\pi (0.52)},0.52\;e^{j2\pi (0.62)}\rangle \right),\\ \left({\hat{a}}_{3},\langle 0.62\;e^{j2\pi (0.72)},0.32\;e^{j2\pi (0.82)}\rangle \right) \end{array}\right\},$$

$$\Psi \left({\hat{℘}}_{4}\right)=\left\{\begin{array}{c} \left({\hat{a}}_{1},\langle 0.52\;e^{j2\pi (0.33)},0.13\;e^{j2\pi (0.42)}\rangle \right),\\ \left({\hat{a}}_{2},\langle 0.34\;e^{j2\pi (0.15)},0.25\;e^{j2\pi (0.16)}\rangle \right),\\ \left({\hat{a}}_{3},\langle 0.46\;e^{j2\pi (0.37)},0.43\;e^{j2\pi (0.48)}\rangle \right) \end{array}\right\}.$$

*Now, for sake of assessing the ambiguous nature of sub-parametric valued tuples, their respective fuzzy parameterised values computed by using Equation (14) are presented in Table 2.*

*In this stage, the opinions and fuzzy parameterised degrees of each decision maker are compiled as FPCIFHSSs and are presented in Table 3, Table 4 and Table 5.*

*Now, all the CIFN-based entries of FPCIFHSSs ${\hat{\Theta }}_{{Dm}_{1}}^{\hat{\Lambda }}$, ${\hat{\Theta }}_{{Dm}_{2}}^{\hat{\Lambda }}$ and ${\hat{\Theta }}_{{Dm}_{3}}^{\hat{\Lambda }}$ are converted to CFNs by using the arithmetical criterion given in Equation (15). The new matrices thus obtained are presented in Table 6, Table 7 and Table 8.*

*In this step, CFN-based entries of matrices $M_{1}^{fpf}$, $M_{2}^{fpf}$ and $M_{3}^{fpf}$ are transformed to fuzzy values first by employing the criterion provided in Equation (16) and then each fuzzy parameterised value corresponding to sub-parametric valued tuples ${\hat{℘}}_{1}$, ${\hat{℘}}_{2}$, ${\hat{℘}}_{3}$ and ${\hat{℘}}_{4}$ is multiplied with the fuzzy valued-based entries in their respective rows. The matrices $M_{1}^{f}$, $M_{2}^{f}$ and $M_{3}^{f}$ thus obtained are presented in Table 9, Table 10 and Table 11.*

*Now, in this phase, the core matrix $M^{core}$ is obtained by the ordinary addition of matrices $M_{1}^{f}$, $M_{2}^{f}$ and $M_{3}^{f}$. Its tabulation formation is provided in Table 12.*

*The score values for the types of brain tumours are computed by taking the average of the entries in their corresponding rows of core matrix $M^{core}$. The score values are presented in Table 13.*

**Figure 8:**
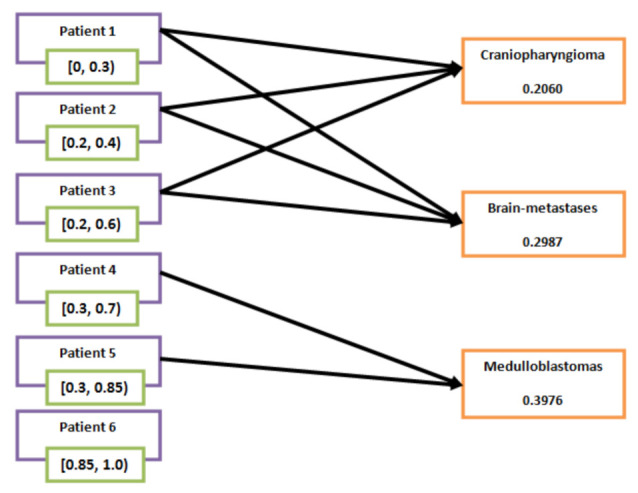
Relation between the patients and brain tumours.

*From Table 13, it is clear that $\hat{\partial }\left({\hat{a}}_{1}\right),\hat{\partial }\left({\hat{a}}_{2}\right)\in \left[0,0.3\right)$, [0.2,0.4),[0.2,0.6) and $\hat{\partial }\left({\hat{a}}_{3}\right)\in \left[0.3,0.7\right),\left[0.3,0.85\right)$; therefore, it is concluded that patients ${\hat{P}}_{1}$, ${\hat{P}}_{2}$ and ${\hat{P}}_{3}$ are suspected to have brain tumours “craniopharyngioma" and “brain-metastases", whereas patients ${\hat{P}}_{4}$ and ${\hat{P}}_{5}$ are suspected to have “medulloblastomas". Patient ${\hat{P}}_{6}$ is outside the scope of the analysis of susceptibility to these three types of brain tumours. This can also be seen in Figure 8.*

## 4. Discussion, Sensitivity Analysis and Comparison

The proposed mathematical model is new and has not been used by any scholar in the literature for the proposed study. Moreover, no relevant literature exists regarding the assessment of analyses of susceptibility of patients to brain tumours by using fuzzy set-like or soft set-like structures. Therefore, the proposed study is not comparable with any existing literature studies based on computational results. However, a comparison is made with its own results by employing different statistical techniques for the determination of scoring values of brain tumours.

As in Example 5, the susceptibility level of patients is assessed by applying the concept of arithmetic mean for determining the score values of brain tumours based on sub-parametric valued tuples and thus five overlapping results and one neutral result are observed. However, the computed scores may vary if other means are applied to find score values that definitely alter the final findings. In this regard, the following cases can be considered:

**Case 1.**  *The score values for the types of brain tumours are computed by taking the geometric means of the entries in their corresponding rows of the core matrix $M^{core}$. The score values are presented in Table 14.*

From Table 14, it is clear that $\hat{\partial }\left({\hat{a}}_{1}\right)=0.2052,\hat{\partial }\left({\hat{a}}_{2}\right)=0.2974\in \left[0,0.3\right),\left[0.2,0.4\right),$[0.2,0.6) and $\hat{\partial }\left({\hat{a}}_{3}\right)=0.3911\in \left[0.3,0.7\right),\left[0.3,0.85\right)$; therefore, it is concluded that patients ${\hat{P}}_{1}$, ${\hat{P}}_{2}$ and ${\hat{P}}_{3}$ are suspected to have brain tumours “craniopharyngioma” and “brain-metastases”, whereas patients ${\hat{P}}_{4}$ and ${\hat{P}}_{5}$ are suspected to have “medulloblastomas”. Patient ${\hat{P}}_{6}$ is outside the scope of the analysis of susceptibility to these three types of brain tumours.

**Case 2.**  *The score values for the types of brain tumours are computed by taking the harmonic means of the entries in their corresponding rows of core matrix $M^{core}$. The score values are presented in Table 15.*

From Table 15, it is clear that $\hat{\partial }\left({\hat{a}}_{1}\right)=0.2044,\hat{\partial }\left({\hat{a}}_{2}\right)=0.2962\in \left[0,0.3\right),\left[0.2,0.4\right),$[0.2,0.6) and $\hat{\partial }\left({\hat{a}}_{3}\right)=0.3854\in \left[0.3,0.7\right)$, [0.3,0.85); therefore, it is concluded that patients ${\hat{P}}_{1}$, ${\hat{P}}_{2}$ and ${\hat{P}}_{3}$ are suspected to have brain tumours “craniopharyngioma” and “brain-metastases”, whereas patients ${\hat{P}}_{4}$ and ${\hat{P}}_{5}$ are suspected to have “medulloblastomas”. Patient ${\hat{P}}_{6}$ is outside the scope of the analysis of susceptibility to these three types of brain tumours.

**Case 3.**  *The score values for the types of brain tumours are computed by taking the harmonic means of the entries in their corresponding rows of core matrix $M^{core}$. The score values are presented in Table 16.*

From Table 16, it is clear that $\hat{\partial }\left({\hat{a}}_{1}\right)=0.2074,\hat{\partial }\left({\hat{a}}_{2}\right)=0.2971\in \left[0,0.3\right),\left[0.2,0.4\right),$[0.2,0.6) and $\hat{\partial }\left({\hat{a}}_{3}\right)=0.3658\in \left[0.3,0.7\right)$, [0.3,0.85); therefore, it is concluded that patients ${\hat{P}}_{1}$, ${\hat{P}}_{2}$ and ${\hat{P}}_{3}$ are suspected to have brain tumours “craniopharyngioma” and “brain-metastases”, whereas patients ${\hat{P}}_{4}$ and ${\hat{P}}_{5}$ are suspected to have “medulloblastomas”. Patient ${\hat{P}}_{6}$ is outside the scope of the analysis of susceptibility to these three types of brain tumours.

**Figure 9:**
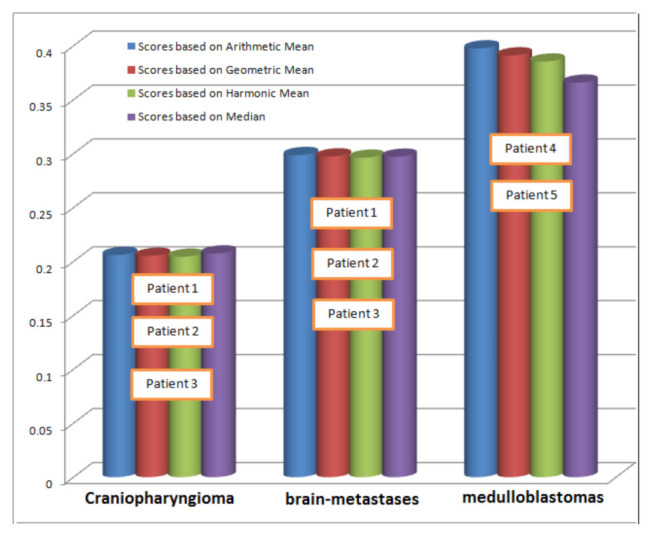
Comparison of scores computed through different statistical techniques.

In all the above cases, the same results are received by applying different Pythagorean means. The combined comparison of scores computed through these cases are presented in Figure 9.

Now, we compare our proposed study with some the most relevant already developed models such as Kumar et al. [34], Papageorgiou et al. [36] and Saeed et al. [37]. This comparison is based on some significant evaluating indicators and is presented in Table 17.

## 5. Conclusions

In the present research, the set-based operations of a flexible arithmetic model FPCIFHSS are used to design an MADM system based on the proposal of a robust algorithm for the assessment of analyses of patients’ susceptibility to brain tumours. This task is accomplished by characterising the basic notions of FPCIFHSS, studying the role of fuzzy parameterisation for the symptoms and their related subcategories, studying arithmetic criteria for the transformation of CIFNs to CFNs and then CFNs to fuzzy values, understanding the process for the selection of parameters and sub-parameters and studying the profiles of decision makers for this evaluation. The suggested method is reliable and flexible, although it has some restrictions on the decision makers’ right to neutral or indefinite membership grades in exchange for their input. The decision makers in the current approach are required to approximatively weigh the alternatives based on their opinions of dependent belonging and non-belonging grades within [0, 1]. When used in a neutrosophic environment, however, where participants can autonomously express their ideas regarding belonging, non-belonging and indeterminate grades within [0, 3], this approach can produce more trustworthy results. Additionally, the information utilised in the form of decision makers’ judgments is fictitious. So it is simple to use this method to discuss a case study that includes actual data from the concerned department. A broad range of research fields, including artificial intelligence, soft computing (fuzzy logic), image processing and classification, pattern recognition and data clustering may fall under the purview of this study. By utilising suitable pseudo-codes through machine learning tools, the proposed algorithm (i.e., the decision support system) can be used further in the classification of brain tumours based on MRI.

## Figures and Tables

**Table 1:** Abbreviations and their full names.

Symbol	Stands for
MCDM	Multi-criteria decision making
MRI	Magnetic resonance imaging
DMP	Decision making problem
FS	Fuzzy set
CFS	Complex fuzzy set
IFS	Intuitionistic fuzzy set
CIFS	Complex intuitionistic fuzzy set
SS	Soft set
HSS	Hypersoft set
MADM	Muti-attribute decision making
FPFHSS	Fuzzy parameterised fuzzy hypersoft set
FPIFHSS	Fuzzy parameterised intuitionistic fuzzy hypersoft set
CFHSS	Complex fuzzy hypersoft set
CIFHSS	Complex intuitionistic fuzzy hypersoft set
CIFN	Complex intuitionistic fuzzy number
CFN	Complex fuzzy number
MAGAF	Multi-argument approximate function
CVF	Complex valued function
AT	Amplitude term
PT	Phase term
FPCIFHSS	Fuzzy parameterised complex intuitionistic fuzzy hypersoft set

**Table 2:** Fuzzy parameterised values corresponding to ${\hat{℘}}_{i},i=1,2,3,4$.

DMs	${\hat{℘}}_{1}$	${\hat{℘}}_{2}$	${\hat{℘}}_{3}$	${\hat{℘}}_{4}$
${Dm}_{1}$	0.15	0.25	0.35	0.45
${Dm}_{2}$	0.51	0.52	0.53	0.54
${Dm}_{3}$	0.54	0.53	0.52	0.295

**Table 3:** Matrix formation of FPCIFHSS ${\hat{\Theta }}_{{Dm}_{1}}^{\hat{\Lambda }}$ constructed with ${Dm}_{1}$.

$M_{1}$	${\hat{a}}_{1}$	${\hat{a}}_{2}$	${\hat{a}}_{3}$
${\hat{℘}}_{1}/0.15$	$\langle 0.12\;e^{j2\pi (0.13)},0.13\;e^{j2\pi (0.14)}\rangle$	$\langle 0.14\;e^{j2\pi (0.15)},0.15\;e^{j2\pi (0.16)}\rangle$	$\langle 0.16\;e^{j2\pi (0.17)},0.13\;e^{j2\pi (0.18)}\rangle$
${\hat{℘}}_{2}/0.25$	$\langle 0.22\;e^{j2\pi (0.23)},0.23\;e^{j2\pi (0.24)}\rangle$	$\langle 0.24\;e^{j2\pi (0.25)},0.25\;e^{j2\pi (0.26)}\rangle$	$\langle 0.26\;e^{j2\pi (0.27)},0.23\;e^{j2\pi (0.28)}\rangle$
${\hat{℘}}_{3}/0.35$	$\langle 0.32\;e^{j2\pi (0.33)},0.33\;e^{j2\pi (0.34)}\rangle$	$\langle 0.34\;e^{j2\pi (0.35)},0.35\;e^{j2\pi (0.36)}\rangle$	$\langle 0.36\;e^{j2\pi (0.37)},0.33\;e^{j2\pi (0.38)}\rangle$
${\hat{℘}}_{4}/0.45$	$\langle 0.42\;e^{j2\pi (0.43)},0.43\;e^{j2\pi (0.44)}\rangle$	$\langle 0.44\;e^{j2\pi (0.45)},0.45\;e^{j2\pi (0.46)}\rangle$	$\langle 0.46\;e^{j2\pi (0.47)},0.43\;e^{j2\pi (0.48)}\rangle$

**Table 4:** Matrix formation of FPCIFHSS ${\hat{\Theta }}_{{Dm}_{2}}^{\hat{\Lambda }}$ constructed with ${Dm}_{2}$.

$M_{2}$	${\hat{a}}_{1}$	${\hat{a}}_{2}$	${\hat{a}}_{3}$
${\hat{℘}}_{1}/0.51$	$\langle 0.21\;e^{j2\pi (0.31)},0.31\;e^{j2\pi (0.41)}\rangle$	$\langle 0.41\;e^{j2\pi (0.51)},0.51\;e^{j2\pi (0.61)}\rangle$	$\langle 0.61\;e^{j2\pi (0.71)},0.31\;e^{j2\pi (0.81)}\rangle$
${\hat{℘}}_{2}/0.52$	$\langle 0.22\;e^{j2\pi (0.32)},0.32\;e^{j2\pi (0.42)}\rangle$	$\langle 0.42\;e^{j2\pi (0.52)},0.52\;e^{j2\pi (0.62)}\rangle$	$\langle 0.62\;e^{j2\pi (0.72)},0.32\;e^{j2\pi (0.82)}\rangle$
${\hat{℘}}_{3}/0.53$	$\langle 0.23\;e^{j2\pi (0.33)},0.33\;e^{j2\pi (0.43)}\rangle$	$\langle 0.43\;e^{j2\pi (0.53)},0.53\;e^{j2\pi (0.63)}\rangle$	$\langle 0.63\;e^{j2\pi (0.73)},0.33\;e^{j2\pi (0.83)}\rangle$
${\hat{℘}}_{4}/0.54$	$\langle 0.24\;e^{j2\pi (0.34)},0.34\;e^{j2\pi (0.44)}\rangle$	$\langle 0.44\;e^{j2\pi (0.54)},0.54\;e^{j2\pi (0.64)}\rangle$	$\langle 0.64\;e^{j2\pi (0.74)},0.34\;e^{j2\pi (0.84)}\rangle$

**Table 5:** Matrix formation of FPCIFHSS ${\hat{\Theta }}_{{Dm}_{3}}^{\hat{\Lambda }}$ constructed with ${Dm}_{3}$.

$M_{3}$	${\hat{a}}_{1}$	${\hat{a}}_{2}$	${\hat{a}}_{3}$
${\hat{℘}}_{1}/0.54$	$\langle 0.24\;e^{j2\pi (0.34)},0.34\;e^{j2\pi (0.44)}\rangle$	$\langle 0.44\;e^{j2\pi (0.54)},0.54\;e^{j2\pi (0.64)}\rangle$	$\langle 0.64\;e^{j2\pi (0.74)},0.34\;e^{j2\pi (0.84)}\rangle$
${\hat{℘}}_{2}/0.53$	$\langle 0.23\;e^{j2\pi (0.33)},0.33\;e^{j2\pi (0.43)}\rangle$	$\langle 0.43\;e^{j2\pi (0.53)},0.53\;e^{j2\pi (0.63)}\rangle$	$\langle 0.63\;e^{j2\pi (0.73)},0.33\;e^{j2\pi (0.83)}\rangle$
${\hat{℘}}_{3}/0.52$	$\langle 0.22\;e^{j2\pi (0.32)},0.32\;e^{j2\pi (0.42)}\rangle$	$\langle 0.42\;e^{j2\pi (0.52)},0.52\;e^{j2\pi (0.62)}\rangle$	$\langle 0.62\;e^{j2\pi (0.72)},0.32\;e^{j2\pi (0.82)}\rangle$
${\hat{℘}}_{4}/0.295$	$\langle 0.52\;e^{j2\pi (0.33)},0.13\;e^{j2\pi (0.42)}\rangle$	$\langle 0.34\;e^{j2\pi (0.15)},0.25\;e^{j2\pi (0.16)}\rangle$	$\langle 0.46\;e^{j2\pi (0.37)},0.43\;e^{j2\pi (0.48)}\rangle$

**Table 6:** Matrix formation of $M_{1}^{fpf}$ with entries in terms of CFNs.

$M_{1}^{\mathrm{fpf}}$	${\hat{a}}_{1}$	${\hat{a}}_{2}$	${\hat{a}}_{3}$
${\hat{℘}}_{1}/0.15$	〈0.005,0.135〉	〈0.005,0.155〉	〈0.015,0.175〉
${\hat{℘}}_{2}/0.25$	〈0.005,0.235〉	〈0.005,0.255〉	〈0.015,0.275〉
${\hat{℘}}_{3}/0.35$	〈0.005,0.335〉	〈0.005,0.355〉	〈0.015,0.375〉
${\hat{℘}}_{4}/0.45$	〈0.005,0.435〉	〈0.005,0.455〉	〈0.015,0.475〉

**Table 7:** Matrix formation of $M_{2}^{fpf}$ with entries in terms of CFNs.

$M_{2}^{\mathrm{fpf}}$	${\hat{a}}_{1}$	${\hat{a}}_{2}$	${\hat{a}}_{3}$
${\hat{℘}}_{1}/0.51$	〈0.05,0.36〉	〈0.05,0.56〉	〈0.15,0.76〉
${\hat{℘}}_{2}/0.52$	〈0.05,0.37〉	〈0.05,0.57〉	〈0.15,0.77〉
${\hat{℘}}_{3}/0.53$	〈0.05,0.38〉	〈0.05,0.58〉	〈0.15,0.78〉
${\hat{℘}}_{4}/0.54$	〈0.05,0.39〉	〈0.05,0.59〉	〈0.15,0.79〉

**Table 8:** Matrix formation of $M_{3}^{fpf}$ with entries in terms of CFNs.

$M_{3}^{\mathrm{fpf}}$	${\hat{a}}_{1}$	${\hat{a}}_{2}$	${\hat{a}}_{3}$
${\hat{℘}}_{1}/0.54$	〈0.05,0.39〉	〈0.05,0.59〉	〈0.15,0.79〉
${\hat{℘}}_{2}/0.53$	〈0.05,0.38〉	〈0.05,0.58〉	〈0.15,0.78〉
${\hat{℘}}_{3}/0.52$	〈0.05,0.37〉	〈0.05,0.57〉	〈0.15,0.77〉
${\hat{℘}}_{4}/0.295$	〈0.195,0.375〉	〈0.045,0.155〉	〈0.015,0.425〉

**Table 9:** Tabular formation of $M_{1}^{f}$ with entries in terms of fuzzy values.

$M_{1}^{f}$	${\hat{a}}_{1}$	${\hat{a}}_{2}$	${\hat{a}}_{3}$
${\hat{℘}}_{1}$	0.0098	0.0113	0.0120
${\hat{℘}}_{2}$	0.0288	0.0313	0.0325
${\hat{℘}}_{3}$	0.0578	0.0613	0.0630
${\hat{℘}}_{4}$	0.0968	0.1013	0.1035

**Table 10:** Tabular formation of $M_{2}^{f}$ with entries in terms of fuzzy values.

$M_{2}^{f}$	${\hat{a}}_{1}$	${\hat{a}}_{2}$	${\hat{a}}_{3}$
${\hat{℘}}_{1}$	0.0791	0.1301	0.1556
${\hat{℘}}_{2}$	0.0832	0.1352	0.3224
${\hat{℘}}_{3}$	0.0875	0.1405	0.1670
${\hat{℘}}_{4}$	0.0918	0.1458	0.1728

**Table 11:** Tabular formation of $M_{3}^{f}$ with entries in terms of fuzzy values.

$M_{3}^{f}$	${\hat{a}}_{1}$	${\hat{a}}_{2}$	${\hat{a}}_{3}$
${\hat{℘}}_{1}$	0.0918	0.1458	0.1728
${\hat{℘}}_{2}$	0.0875	0.1405	0.1670
${\hat{℘}}_{3}$	0.0832	0.1352	0.1612
${\hat{℘}}_{4}$	0.0266	0.0163	0.0605

**Table 12:** Tabular formation of core matrix $M^{core}$.

$M^{\mathrm{core}}$	${\hat{a}}_{1}$	${\hat{a}}_{2}$	${\hat{a}}_{3}$
${\hat{℘}}_{1}$	0.1807	0.2872	0.3404
${\hat{℘}}_{2}$	0.1995	0.3070	0.5219
${\hat{℘}}_{3}$	0.2285	0.3370	0.3912
${\hat{℘}}_{4}$	0.2152	0.2634	0.3368

**Table 13:** Scores of alternatives ${\hat{a}}_{i},i=1,2,3$ corresponding to ${\hat{℘}}_{j},j=1,2,3,4$.

Brain Tumour Types	$\hat{\partial }\left({\hat{a}}_{i}\right)$: Score Values of ${\hat{a}}_{i},i=1,2,3$
${\hat{a}}_{1}$	0.2060
${\hat{a}}_{2}$	0.2987
${\hat{a}}_{3}$	0.3976

**Table 14:** Scores of alternatives ${\hat{a}}_{i},i=1,2,3$ corresponding to ${\hat{℘}}_{j},j=1,2,3,4$ computed through geometric mean.

Brain Tumour Types	$\hat{\partial }\left({\hat{a}}_{i}\right)$: Score Values of ${\hat{a}}_{i},i=1,2,3$
${\hat{a}}_{1}$	0.2052
${\hat{a}}_{2}$	0.2974
${\hat{a}}_{3}$	0.3911

**Table 15:** Scores of alternatives ${\hat{a}}_{i},i=1,2,3$ corresponding to ${\hat{℘}}_{j},j=1,2,3,4$ computed through harmonic mean.

Brain Tumour Types	$\hat{\partial }\left({\hat{a}}_{i}\right)$: Score Values of ${\hat{a}}_{i},i=1,2,3$
${\hat{a}}_{1}$	0.2044
${\hat{a}}_{2}$	0.2962
${\hat{a}}_{3}$	0.3854

**Table 16:** Scores of alternatives ${\hat{a}}_{i},i=1,2,3$ corresponding to ${\hat{℘}}_{j},j=1,2,3,4$ computed through median.

Brain Tumour Types	$\hat{\partial }\left({\hat{a}}_{i}\right)$: Score Values of ${\hat{a}}_{i},i=1,2,3$
${\hat{a}}_{1}$	0.2074
${\hat{a}}_{2}$	0.2971
${\hat{a}}_{3}$	0.3658

**Table 17:** Structural comparison of proposed study with the most relevant existing model.

References	Entitlement of Fuzzy Parameterisation	Handling of Information-Based Periodicity	Provision of Susceptibility-Based Ranking for Brain Tumours	Pythagorean Means-Based Sensitivity Analysis of Results
Kumar et al. [34]	Inadequate	Inadequate	Inadequate	Inadequate
Papageorgiou et al. [36]	Inadequate	Inadequate	Inadequate	Inadequate
Saeed et al. [37]	Inadequate	Inadequate	Inadequate	Inadequate
Proposed model	Parameters and sub-parameter-based uncertainties are managed by using the concept of fuzzy parameterisation	Information-based periodicity is tackled by using amplitude and phase values in complex plane settings	Patients and types of brain tumours are matched and ranked in accordance with the susceptibility scores	Sensitivity of computed scores is observed through employing the formulations of Pythagorean means (i.e., arithmetic mean, geometric mean and harmonic mean)

## Data Availability

Not applicable.
